# Design and manufacture of an ultra-compact, 1.5 T class, controlled-contact resistance, REBCO, brain imaging MRI magnet

**DOI:** 10.1088/1361-6668/ad80d5

**Published:** 2024-10-15

**Authors:** B Parkinson, K Bouloukakis, H W Weijers, J Olatunji, M Szmigiel, M W Hunter, T Froelich, J Bailey, M Garwood

**Affiliations:** 1 Robinson Research Institute of Victoria University of Wellington, Wellington, New Zealand; 2 OpenStar Technologies LTD, Wellington, New Zealand; 3 Air Liquide SA, Paris, France; 4 Transpower LTD, Wellington, New Zealand; 5 Center for Magnetic Resonance Research, University of Minnesota, Minneapolis, MN, United States of America

**Keywords:** high temperature superconductor, MRI, no-insulation coils, neuro-imaging

## Abstract

Brain imaging MRI comprises a significant proportion of MRI scans, but the requirement for including the shoulders in the magnet bore means there is not a significant size reduction in the magnet compared to whole-body magnets. Here we present a new design approach for brain imaging MRI magnets targeting ±20 kHz *B*_0_ variation over the imaging volume rather than the more usual ±200 Hz making use of novel high-bandwidth MRI pulse sequences and distortion correction. Using this design approach, we designed and manufactured a 1.5 T class ReBCO cryogen-free magnet. The magnet is dome-like in form, completely excludes the shoulders and is <400 mm long. The magnet was wound using no-insulation style coils with a conductive epoxy encapsulant where the contact resistance of the coils was controlled so the emergency shut-down time of the magnet was less than 30 s. Despite acceptable coil testing results ahead of manufacture, during testing of the magnet, several of the epoxy coils showed signs of damage limiting stable performance to <55 A compared to the designed 160 A. These coils were replaced with insulated paraffin encapsulated coils. Subsequently the magnet was re-ramped and was stable at 81 A, generating 0.71 T as several other coils had sustained damage not visible in the first magnet iteration. The magnet has been passive shimmed to ±20 kHz *B*_0_ variation over the imaging volume and integrated into an MRI scanner. The stability of the magnet has been evaluated and found to be acceptable for MRI.

## 1. Introduction

With brain and head imaging comprising a significant proportion of MRI scans, many groups have considered designs for head imaging magnets [1–5]. However, it is typically not possible to cost-effectively construct a conventional superconducting MRI magnet whose length is less than 1.2 times the bore diameter [6]. The practical implication of this design rule is the patient’s shoulders remain inside the magnet. As such, there are only marginal gains in magnet volume to be had from targeting a head-sized imaging volume rather than a whole-body sized imaging volume.

Recent developments in MRI pulse sequences [7, 8] have demonstrated it is possible to achieve clinical quality imaging in magnetic field (*B*_0_) having >100 kHz of variation in proton precession frequency over the imaging volume rather than the usual 100’s Hz of variation typical in conventional MRI scanners. By targeting a 1.5 T magnet designed for brain scanning with ∼±20 kHz *B*_0_ variation over the imaging volume, it may now be possible to reduce the length of the magnet to be comparable with the bore diameter, allowing the shoulders to be completely excluded, yet retain the ability to achieve clinical quality imaging.

In parallel, progress in privately funded nuclear fusion projects [9, 10] means there is now a real volume market for high-performance ReBCO conductors in the foreseeable future. As such, it is likely costs of ReBCO conductor will reduce substantially. We therefore chose to manufacture the magnet from ReBCO to demonstrate possible features of a ReBCO magnet anticipating its more widespread use in magnets. Critically, using ReBCO conductor means the cryogenics of the magnet may become substantially simplified compared to conventional recondensing clinical MRI magnets [6].

We investigate whether it is possible to use a high-capacity single stage pulse-tube cryocooler to cool the magnet, with the cryocooler fulfilling both the magnet and current lead cooling duties. Since the magnet only has one cooling source, the radiation shield is eliminated, which in turn eliminates a major source of eddy currents arising from the interaction of the magnet with the gradient coils. Using a single stage cryocooler means magnet cool down times can be minimised compared to more conventional cryogen-free magnet. An emphasis is therefore placed on minimising the thermal mass of the magnet components to minimise cool down time, whilst also reducing the eddy current response of the magnet components to minimise interaction with the gradient coil.

ReBCO magnets are intrinsically extremely unlikely to quench during operation. However, consideration of a potential quench protection solution for ReBCO magnets is required. Here, we discuss the use of no-insulation style coils to provide this protection, including the use of an external dump resistor used in conjunction with no-insulation coils. Emergency shut-down of the magnet is a critical design parameter, typically requiring the magnet to ramp from full field to near zero in <30 s [11]. Since the contact resistance of the coils ultimately determines the upper bound for magnet shut-down time, the contact resistance must be chosen such that the magnet can be ramped down in no more than the required time. Previously the design of a filled epoxy resin system to allow tuneable contact resistance between turns whilst maintaining close control over the coil sizing has been explored [12]. Here we design a variant of the epoxy resin system for this particular application.

At present, MRI scanners are seldom found outside of large urban hospitals due to their high purchase price, infrastructure requirements, high installation cost and considerable operational expenses [13]. Here, we describe the design and manufacture of an ultra-compact 1.5 T class brain imaging magnet. It is hoped the resulting MRI scanner will be a stepping-stone to address some of the existing impediments to MRI accessibility. The magnet is a key component of a grant to build an MRI system with clinical quality 1.5 T imaging, but with lower magnetic field uniformity than conventional clinical MRI magnets.

## 2. Field uniformity and conductor positioning

The first step of the design process for these magnets is to define the regions in which coils may be placed. Since this is for a proof of principal system, we have decided to make the magnet warm bore relatively compact to reduce the overall size of the system. There is no need for a head or brain imaging magnet to have access from both ends, resulting in the decision to offset the imaging volume from the geometric centre of the magnet towards the patient end. Similarly, we decided to taper the bore at the other end of the magnet to minimize conductor usage. Since this magnet is designed as a brain imaging, rather than whole-head imaging magnet, we chose an elliptical imaging volume 200 mm in the major (radial) axis and 150 mm in the minor (axial) axis.

**Figure 1.**
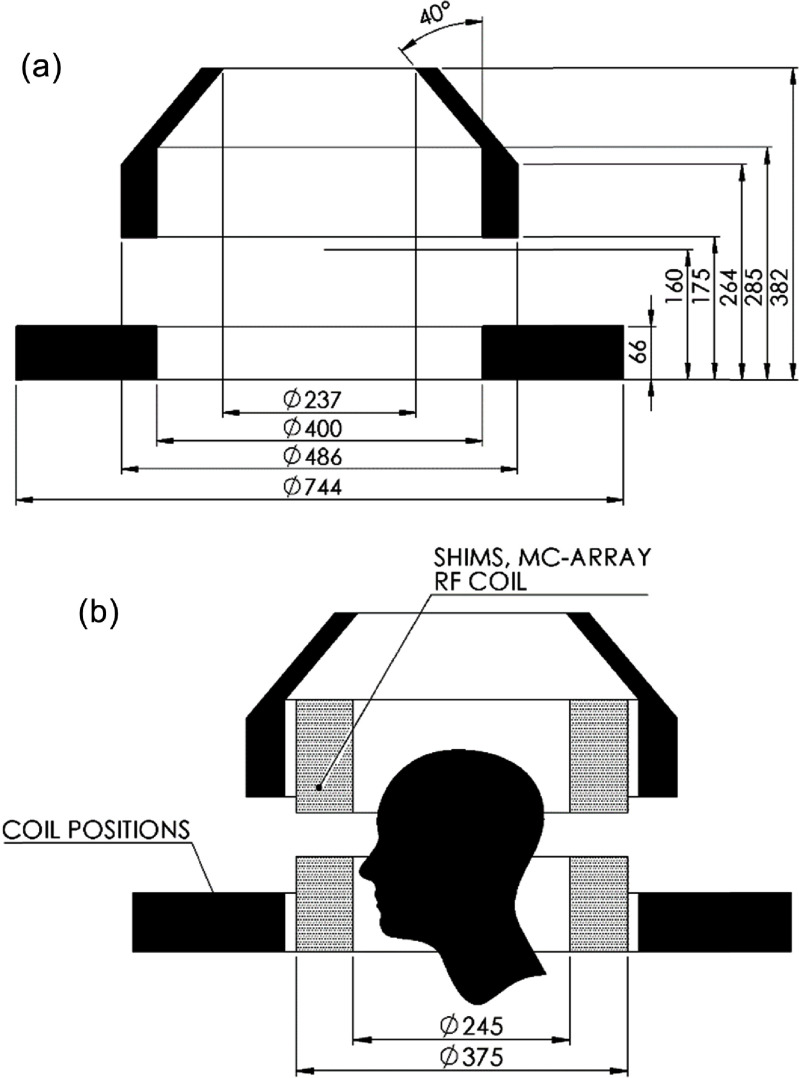
(a): Diagram illustrating the space available for conductors (in black) to meet the ergonomic requirements of the magnet (all dimensions in mm). (b): Overlay of head inside available space along with space for the passive shim cassette, multi-coil array and radio-frequency coil.

The warm bore of the magnet is a ⌀375 mm cylinder at the patient end (figure 1). The cylindrical portion of the warm bore extends 290 mm axially from the patient end. At this point, the magnet bore tapers in at an angle of 40° from vertical to form a cone-like shape, reducing to a ⌀125 mm opening at the opposite end of the magnet located 440 mm from the patient end. This rather narrow magnet bore compared to imaging volume size is in part enabled by the use of an unshielded gradient coil solution which has very low radial thickness.

The isocenter is positioned 175 mm axially distant from the patient end of the magnet warm bore. From anthropometric data [14], such an isocenter position enables approximately 95% of the adult population to be scanned with their brain positioned in the imaging volume and their shoulders excluded from the magnet. Practically, this means the magnet coils must now be positioned no further than 160 mm below the isocenter to allow space for the magnet cryostat, insulation and coil mechanical support. Similarly, there is a 15 mm space allowance between the internal diameter (ID) of the magnet warm bore and the ID of the superconducting coils.

Since the patient space inside the magnet is extremely compact, we designed a window through the side of the magnet to help reduce patient claustrophobia. The window is positioned to allow approximately 95% of the adult population to see through the window when the top of their skull is located 100 mm above the top of the imaging volume [14]. The top of the window is at the same height as the isocenter, and the bottom of the window is located 70 mm below the isocenter, and the window is 180 mm wide. Again, 15 mm is allowed between the room temperature window surfaces and coil positions.

Collating these dimensions, possible locations of the coils are shown in figure 1 in the shaded areas, with a maximum coil diameter of ø 744 mm. The possible conductor locations are then discretized into a series of pixelated windings, each of which has identical radial and axial extent, and each of which has equal current density, here chosen to be 200 A mm^−2^. Since this magnet will be manufactured from 4 mm wide high temperature superconductor (HTS) tapes, we expect to wind the magnet coils as a series of double-pancake coils. The axial and radial extent of a winding pixel is hence chosen as 4 × 4 mm respectively. Insulating disks bring the double-pancake axial thickness to 8.8 mm and cooling disks between double pancakes bring the axial discretization of double pancake coil positions to 9.6 mm.

## 3. Magnet optimisation

Following the method of [15], the ellipsoidal harmonics of *B_z_* (in tesla) relative to 1.5 T are calculated up to 10th order for each of the possible conductor locations. A look up table, *S*, is subsequently generated for *B_z_* (in tesla) at the isocenter up to the total number of coils (nc), and each *A_n,_*_0_ (in tesla) up to *n* = 4 for each of the pixel-like windings. Since the magnet is not symmetric, odd order terms must be considered as well as even order terms,

\begin{equation*}S = \left[ {\begin{array}{*{20}{c}} {\begin{array}{*{20}{c}} {B_z^1}&amp;{A_{1,0}^1}&amp; \ldots &amp;{A_{4,0}^1} \\ {B_z^2}&amp;{A_{1,0}^2}&amp; \ldots &amp;{A_{4,0}^2} \\ \vdots &amp; \vdots &amp; \vdots &amp; \vdots \\ {B_z^{{\text{nc}}}}&amp;{A_{1,0}^{{\text{nc}}}}&amp; \ldots &amp;{A_{4,0}^{{\text{nc}}}} \end{array}} \end{array}} \right].\end{equation*}

Linear programming is then used (MATLAB, Mathworks, USA) to solve:

\begin{equation*}\mathop {\min }\limits_{{I_{{\text{dens}}}}} 2\pi {r_c}^T{\text{such that}}\left\{ \begin{array}{*{20}{c}} {S.{I_{{\text{dens}}}} = {\text{ }}{s_{{\text{target}}}}} \\ { - 1 &lt; {I_{{\text{dens}}}} &lt; 1} \end{array}\right.\end{equation*}

where 2π*r_c_^T^* is the transpose of an ordered list of circumferences at the centroid of each of the nc pixel-like conductors, *I*_dens_ is the corresponding current density of each of the nc pixel-like conductors where *I*_dens_ = 1 corresponds to a current density of 200 Amm^−2^, and *s*_target_ is the list of target harmonics, where $s = \left[ {1.5,{\text{ }}0,0,0,0} \right].$

We have used 2π*r_c_* as the function to minimize the amount of conductor.

Linear programming principally identifies which axial double pancake coil positions are required and the radial dimensions of these coils. Inevitably, some relative pixel current densities at coil edges fall above −1 or below 1. A simple approach is to round these pixel values to their nearest integer value; however, this discretization necessarily reduces the uniformity achieved in the linear programming step.

**Figure 2.**
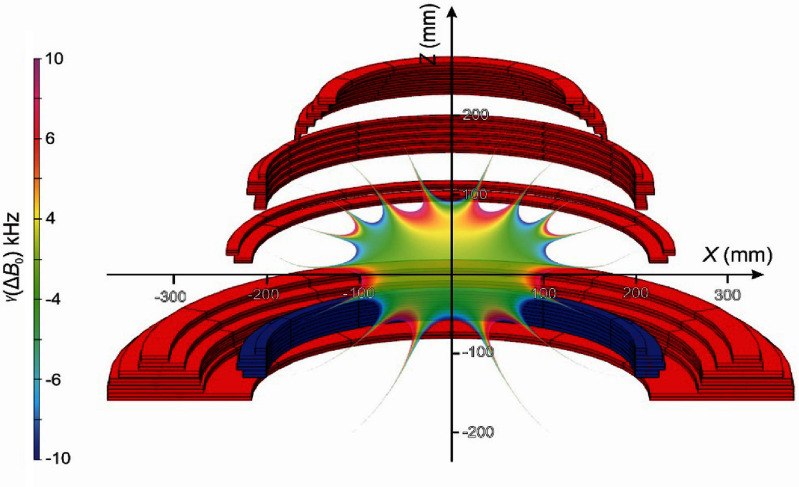
Final magnet geometry showing cross-sectional view of the windings and the designed high-uniformity area. Red coils are designed with current flow in a clockwise direction; blue coils have the opposite polarity.

To improve uniformity following discretization, a MATLAB implementation of the Nelder–Mead method [16] is used to iteratively adjust the coil inner and outer radii found from the linear programming step, subject to the coil dimensions not exceeding the available space. The axial positions of the coils are fixed to avoid coil overlap during optimization. Rather than target ellipsoidal harmonics in the second optimization step, we instead explore minimizing peak-to-peak variation in *B_z_* at a series of eight points distributed at Gauss angles on the surface of the 200 mm × 150 mm ellipse. *B_z_* is the component of field evaluated at each of the *I* points along the ellipse. The cost function for the Nelder–Mead algorithm to achieve a 1.5 T magnet is then given by:

\begin{equation*}{\text{cost}} = \mathop \sum \limits_{i = 1}^8 {c_i}\left| {{B_{{z_i}}} - 1.5} \right| + { }{c_9}{l_{{\text{wire}}}},\end{equation*}

where each field evaluation point *B_zi_* is weighted by *c_i_* for each of the *i* field measurement points. It is possible to use the Gauss weight for *c_i_* since the points have been arranged by their Gauss angles; however, we found it more effective to simply set *c_i_* = 1 since the points are not being used to calculate harmonics of any kind. *l*_wire_ is the total length of tape used to manufacture the particular magnet iteration, and *c*_9_ is the weight to bring the wire length to the same order of magnitude as the field error terms. Alternatively, a particular wire length may instead be targeted. From this starting point, we have designed a magnet with ±20 kHz peak-to-peak field variation over a 150 × 200 mm ellipsoidal (i.e. brain shaped) imaging volume (figure 2). By reducing the uniformity of the field, the magnet can now be made significantly shorter than conventional design practice allows, meaning the patient’s shoulders can be completely excluded from the bore of the magnet.

## 4. Determination of magnet operating point and superconductor selection

With the coil positions and dimensions finalised, we calculated the field and field angle on the conductors. This was done by subdividing each of the coils into approximately 1 mm pixel-like sections in the radial direction and calculating the field and field angle at the centroid of each of these pixel-like sections. The result of this calculation is shown in figure 14 (upper and lower left), with peak field on the windings of 5.1 T at a current density of 200 A mm^−2^. We chose to operate the magnet at 160 A to achieve 1.5 T central field at a suitable turn density. An explanation for the relatively low operating current will be provided in the cryogenic design section (section 6) and the radial build of each turn in section 9.

To determine the operating temperature and hence operating point of the magnet relative to *I_c_*, we measured a series of 6 samples of YGdBCO conductor using an automated characterisation tool [17]. Characterisation of the superconductor was performed at 24, 26, 28 and 30 K. We then divided the in-field, low temperature *I_c_* by the 77 K self-field *I_c_* to determine the ‘lift factor’. From these and other samples from this particular supplier, we have found the 77 K self-field *I_c_* measurement alone is able to predict the low temperature in-field *I_c_* with approximately a 10% error. This result gave us confidence we could order superconductor with reasonable confidence in the in-field, low temperature *I_c_* from the 77 K *I_c_*. A four-dimensional look-up table was created for the critical current data (i.e. field, field-angle, temperature and critical current). Linear interpolation was then used to index the look-up table to calculate critical current everywhere in the magnet when operated at 30 K, assuming conductor with a constant 77 K self-field *I_c_* everywhere in the magnet. Since superconductor cost tends to scale with 77 K *I_c_*, we calculated the minimum 77 K *I_c_* required in the magnet to support operation at less than 70% of critical current everywhere. The minimum *I_c_* for each coil is shown in table 1. The temperature dependence of *I_c_* also illustrates that the choice of operating current of the magnet is intimately linked to the maximum allowable operating temperature of the magnet at full field.

**Table 1.** Nominal design parameters.

Coil	Coil pack	Coil ID (mm)	Coil OD (mm)	# turns (−)	Type	Min *I_c_* (77 K, SF) (A)	Tape width (mm)	Tape length (m)
1	1	432	744	1561	Eu	125	4	2889
2	1	535	744	1043	YGd	130	4	2102
3	1	543	740	985	YGd	130	4	1991
4	2	400	451	−255	YGd	150	4	346
5	1	545	720	878	Eu	125	4	1750
6	2	400	464	−320	YGd	125	4	439
7	1	576	677	502	Eu	125	4	992
8	2	400	459	−295	YGd	130	4	402
9	1	611	672	302	Eu	125	4	613
10	2	400	424	−122	YGd	130	4	163
11	3	431	470	198	YGd	130	3	285
12	3	461	486	129	YGd	130	3	196
13	4	400	427	133	YGd	130	3	178
14	4	400	424	121	YGd	130	3	162
15	4	400	442	209	YGd	130	3	282
16	4	400	440	201	YGd	140	3	270
17	4	400	417	83	YGd	150	3	111
18	5	328	339	53	YGd	130	3	60
19	5	312	337	126	YGd	130	3	134
20	5	296	331	175	YGd	130	3	177
21	5	280	312	161	YGd	130	3	155
22	5	264	300	182	YGd	130	3	166
23	5	248	288	199	YGd	130	4	172

Here, ID: Inner Diameter; OD: Outer Diameter; Min *I_c_*(77 K, SF): Minimum critical current at 77 K, self-field conditions. If a coil is intended to have a negative polarity relative to the rest of the coils, the number of turns is marked with a negative sign. The magnet uses a total of 14 km of conductor.

There is a large range in the minimum required critical current. To reduce conductor cost and to better balance the critical current margin across the magnet, we decided to purchase 3 mm conductor for coils with the lowest critical current rating. By contrast, coils with the largest critical current rating exceeded the performance of routinely available YGdBCO conductor at the time. At that time, the superconductor vendor introduced a new ReBCO formulation—namely EuBCO which was designed for improved in-field performance. As for the YGdBCO samples, we measured a series of 5 EuBCO samples using the same characterisation system. Lift factors were once again readily predictable from the 77 K self-field *I_c_*. For the coils requiring minimum conductor *I_c_* exceeding 170 A using YGdBCO conductor, we instead used EuBCO conductor. The final superconductor specification (type) and minimum required *I_c_* is shown in table 1 and should allow for safe operation of the magnet at temperatures up to 30 K.

## 5. Magnet cryogenic design

A use case for the MRI scanner is to operate at a series of remote clinics, where the magnet is warmed up, transported to the new site, and then cooled down again ahead of use. As such, the magnet is cryogen-free for ease of transportation and engineered for fast cool-down. The magnet is designed using a single-stage Cryomech PT-93 pulse-tube cryocooler to rapidly cool the magnet. Usually cryogen-free magnets use two-stage cryocoolers. In that case, the first stage is used to cool the copper current leads from the outside world and the thermal shield if fitted. The second stage is used to cool the magnet and the HTS current leads from the first stage to the operating temperature of the magnet. Since this magnet is cooled with a single stage cryocooler, the single stage of the cryocooler must do both the jobs usually performed by a two-stage cryocooler. This solution provides faster cool down to the operating temperature at the expense of a higher minimum temperature achievable by the cryocooler.

The magnet is designed without a thermal shield to minimise magnet volume and reduce the eddy current interaction between the magnet and the unshielded gradient solution, relying exclusively on multi-layer insulation for thermal radiation rejection. Moreover, since the magnet is cooled with a single-stage cryocooler there is not a heat sink available for the thermal shield without adding an additional cooling element. Without a thermal shield, there will be considerable heat flow through the magnet from the residual thermal radiation not rejected by the multi-layer insulation.

**Figure 3.**
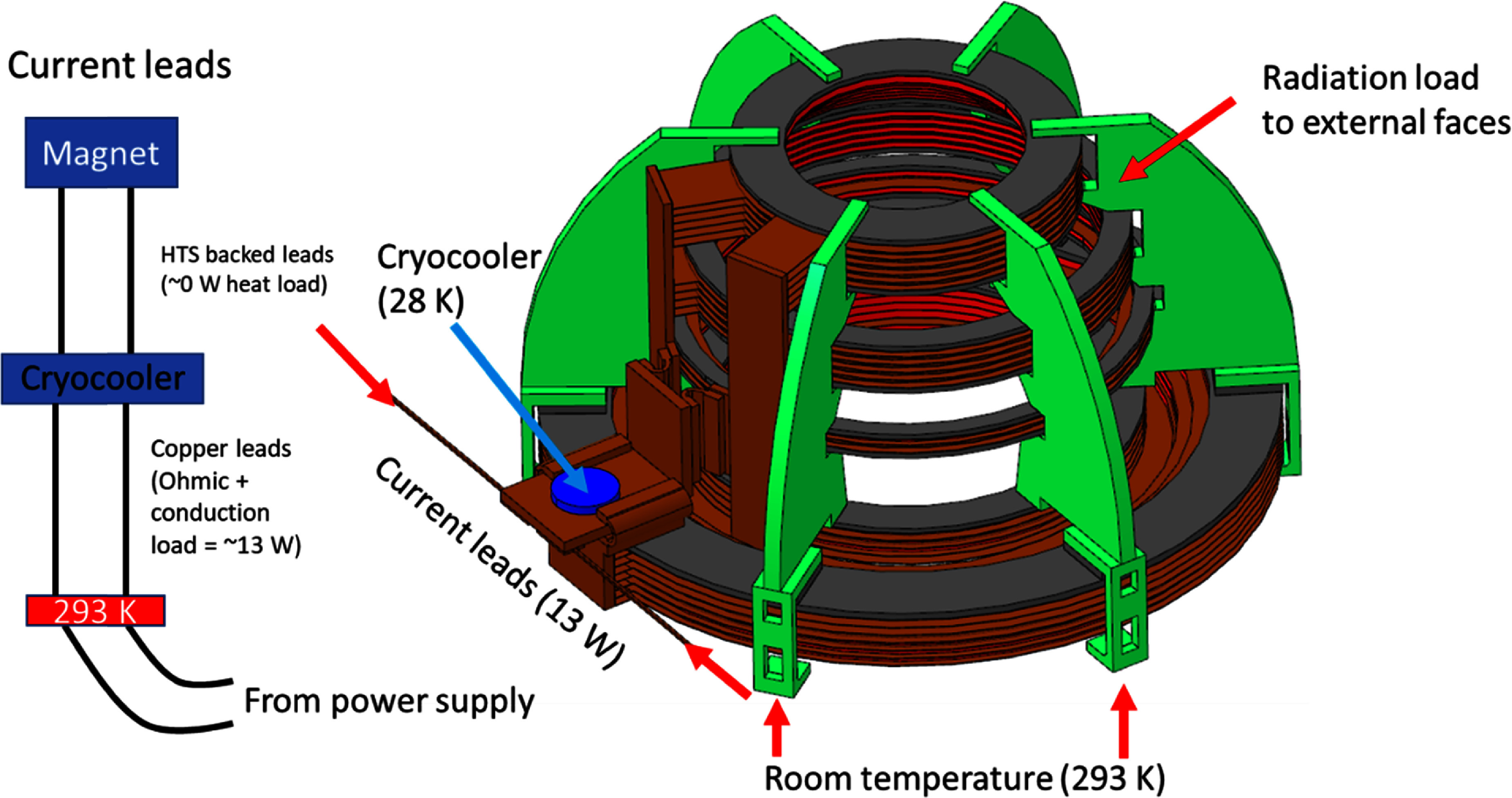
Cryogenic features of the magnet structure.

As can be seen in figure 3, each double-pancake coil is sandwiched between two thin (0.8 mm) copper cooling plates. The copper cooling buses were slit to reduce eddy currents during ramping and upon switching of the MRI gradient system. These copper cooling plates are in turn terminated onto one of three cooling buses. The cooling buses are finally connected to the cryocooler itself. The magnet is supported on a series of six fiberglass strut-like pieces. These struts not only act as spacers for the five coil packs but also position the magnet inside the cryostat. We created a 3D static thermal simulation of the magnet using the Opera-3D (Dassault Systèmes, France) finite element modelling environment as shown in figure 3. The surface representing the cryocooler in the model is set to be a fixed temperature (28 K) in the model. Non-linear thermal conductivities were used for the constituent materials [18]. The base of the strut elements is set to be 300 K—i.e. room temperature. It is conservatively assumed there is a thermal conductivity of 5 × 10^−3^ Wm^−2^K^−1^ between any adjacent surfaces where heat must flow from one body to the next (i.e. thermal interface surface) [18] and all external surfaces experience a radiation load of 2.2 Wm^−2^.

**Figure 4.**
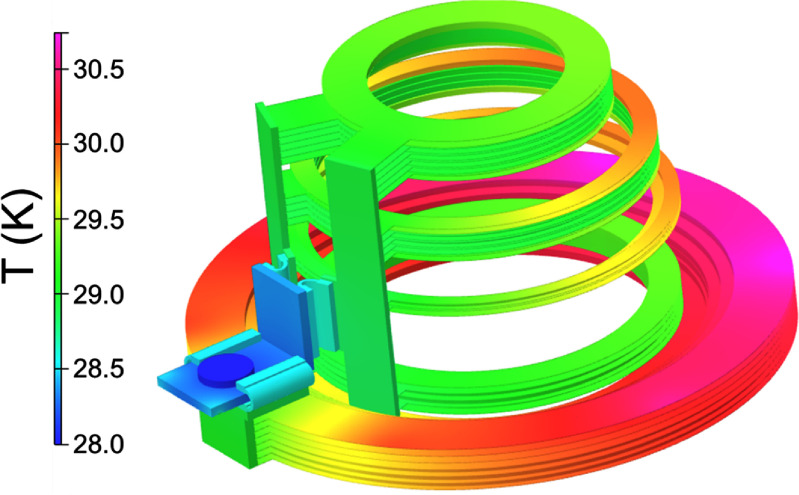
Temperature distribution in the magnet as designed.

The result of the simulation is shown in figure 4. To support this temperature distribution, the cryocooler must extract 8.2 W at zero field. Additional cooling power is required to cool the current lead terminations, as discussed below.

**Figure 5.**
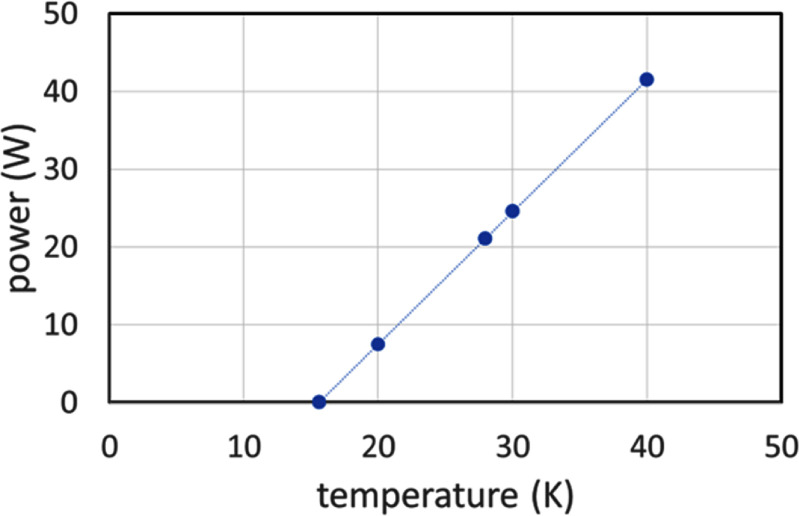
Detail of the PT-93 cryocooler capacity curve.

We chose a pulse-tube cryocooler to minimise vibration into the magnet thereby reducing the possibility of MRI motion artefacts. The Cryomech PT-93 cryocooler is a low-temperature derivative of the Cryomech PT-90. The PT-93 cryocooler capacity curve is shown in figure 5.

For the coils to operate at no more than 30 K, the cryocooler must operate at no more than 28 K at full field. At 28 K and when operating with a 60 Hz electrical supply, the cryocooler can remove removing 23 W of heat. This, together with the 8.2 W to cool the magnet, limits the allowable heat load from the current leads. For optimised current leads, the heat load per lead is 42 mW per lead per ampere [19] corresponding to 13.4 W total heat load at 160 A. The total designed heat load is therefore 21.4 W with 23 W cooling available, allowing operation of the magnet at 160 A.

## 6. Magnet structure and static forces

**Figure 6.**
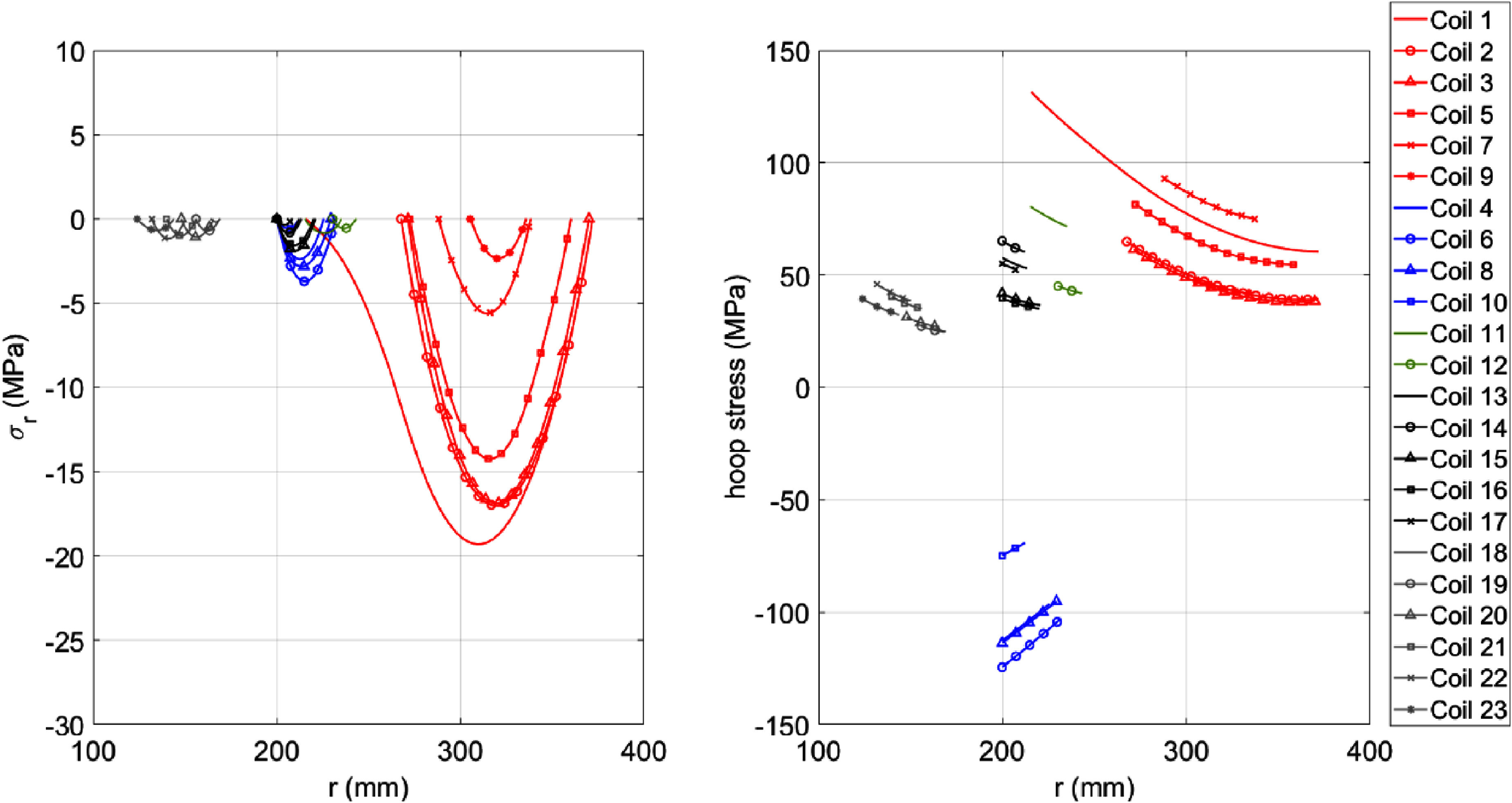
Calculated radial (left) and hoop stress (right) assuming the coil outer diameters are rigidly constrained. The coils are grouped into 5 packs delineated by colour. Here, stress is plotted against the coil radial dimensions.

We created a 2D magnetostatic model of the magnet in the Opera-2D FE package (Dassault Systèmes, France). From this magnetostatic model of the magnet, it is possible to calculate the Lorentz forces. It is presumed the axial Lorentz forces between coils can be used directly as an external force in a mechanical model. However, the radial Lorentz forces within a coil must be converted to hoop and radial stresses via a suitable model. Here, we created an analytical stress model adapting earlier work [20]. We also cross-referenced the analytical model against an FE model in Opera-2D using the Lorentz forces from the magnetostatic model as an input to a static stress model assuming the composite coil is copper-like with indicative Poisson ratio of 0.31 and Young’s Modulus of 114 GPa. Assuming the coil has floating boundary conditions on either end, the resulting radial stress and hoop stress for the magnet coils are presented in figure 6.

These values are comparable with typical MRI magnets [21] and do not cause unusual concerns. However, coils 4, 6, 8 and 10 have opposite polarity to the remainder of the coils and hence have a negative (i.e. compressive) hoop stress. The coils were designed with a titanium bobbin to prevent the coils collapsing inwards under the compressive hoops stress.

In addition to the radial stresses within coils, the axial stresses between coils were considered. These stresses are managed by the coil support elements and in turn by the spacing struts shown earlier in the thermal design. Table 2 shows the axial forces on each double pancake. These forces are then applied to a mechanical finite element model of the supporting structure in SolidWorks (Dassault Systèmes, France). Titanium was used for the coil supports, with G10 used for the arcs spacing the coil packs apart. G10 was used for its low thermal conductivity and low thermal mass. Titanium was used as a low weight, but relatively thermally conductive material. It minimised the cool down time of the magnet compared to composite or stainless-steel alternatives. Aluminium was not chosen due to its high electrical conductivity and hence likely high contribution to eddy currents from gradient switching.

**Table 2.** Peak Von Mises axial force and hoop stress in the magnet as designed.

Coil	Axial force (MPa)	Hoop stress (MPa)
1	3.6	212
2	2.2	252
3	0.1	267
4	2.8	−31
5	−1.8	264
6	1.6	−21
7	−3.4	224
8	0.2	−20
9	−4.6	130
10	−0.8	−27
11	0.5	126
12	−0.4	97
13	1.3	113
14	0.6	123
15	0.2	126
16	−0.9	119
17	−1.6	101
18	0.8	54
19	0.7	72
20	0.2	80
21	0.0	84
22	−0.4	87
23	−1.2	73

**Figure 7.**
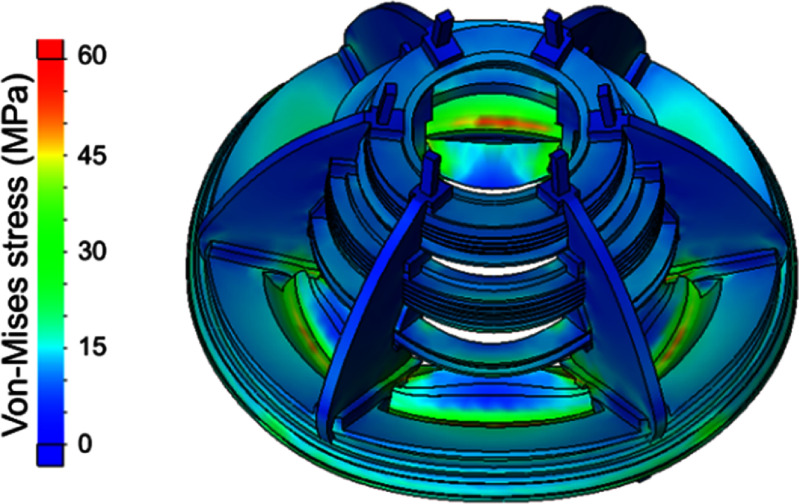
Von-Mises stress in the cold mass at full field as designed.

**Figure 8.**
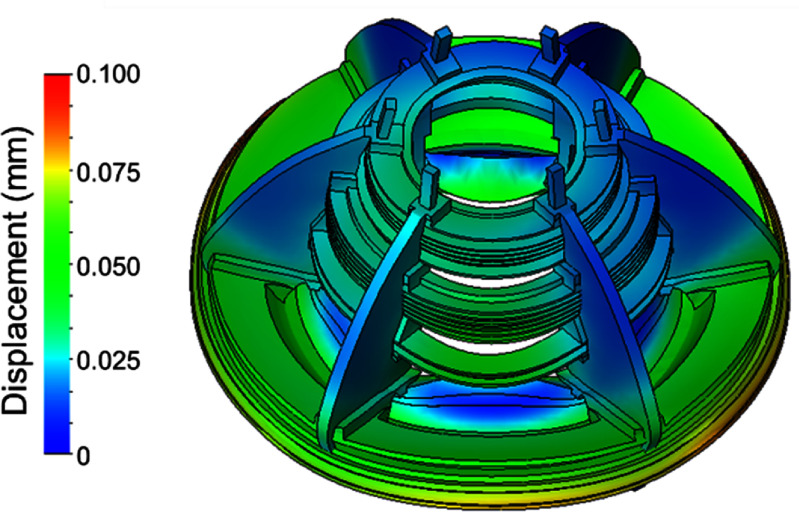
Magnitude of displacements in the cold mass at full field as designed.

The resulting Von-Mises stresses and magnet deflection at full field are shown in figures 7 and 8 respectively. The peak deflection of coils within the magnet is 0.1 mm, conservatively assuming the coils have negligible self-strength in the axial-direction. All materials are far from their yield point.

## 7. Quench protection

### 7.1. No insulation coil considerations

Traditionally, superconducting coils are wound with an insulating material between coils. However, it was found it is possible to wind HTS coils without insulation between turns [22]. At the time the project was proposed in 2016, there was great interest in such no-insulation style coils [23–28], as they showed evidence of being self-protecting in the event of quench. However, the quench protection comes at the expense of long ramping and switching times for the magnet [22].

Since we intend to use this MRI magnet clinically, it is critical the magnet meets usual clinical guidelines for safe usage. Specifically, the magnet’s field needs to be switched to near-zero in no more than 30 s in case of an emergency [11]. We have interpreted this as meaning the field strength must be <50 mT after 30 s. As an HTS magnet, we chose to operate the magnet in the driven mode. To activate the emergency mode, the power supply is disconnected from the magnet using a contactor and the magnet’s stored energy is dissipated inside the magnet’s coils. In this scenario, the conductive path between turns of the coils creates an effective resistance for the magnet. The emergency ramp-down time of the magnet is therefore set by the L–R time constant, i.e. the ratio of the magnet’s inductance (*L*) and the effective resistance. Since we need a time constant of ∼5 s, this sets a minimum value for the effective resistance of the magnet and hence the resistance between turns.

Previous studies have described a derivative of the no-insulation coil method to quench protect magnets with control over contact resistivity. In this study, an epoxy system is described [12]. The epoxy is filled with uncoated diamonds to precisely gauge the gap between adjacent turns and copper particles are added to create electrical conductivity to the epoxy. The epoxy system is then used as a wet-wound encapsulant in a REBCO coil co-wound with a 80 *µ*m titanium tape. Titanium is used as the metallic co-wind for its mechanical properties and low heat capacity. Now, we need to define the target contact resistivity needed for this particular magnet.

### 7.2. Modelling of the no-insulation coils

**Figure 9.**
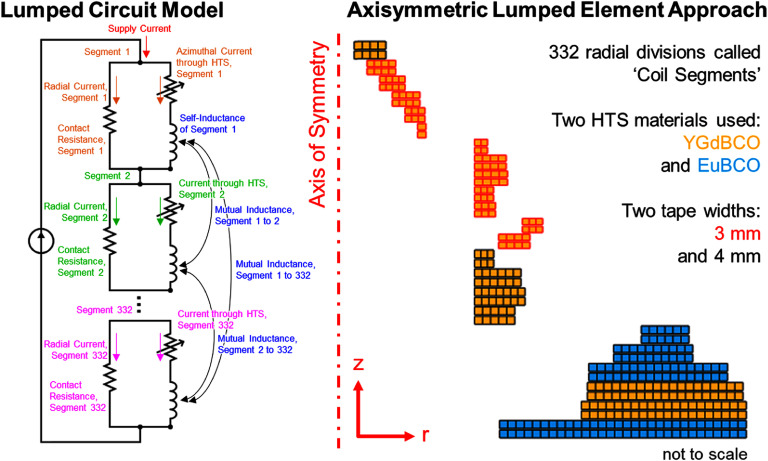
NI quench model set up. The two different ReBCO formulations are used in different sections of the magnet to utilise the superior in-field performance of the EuBCO conductor in critical areas of the magnet. The different performance of the two conductor types form part of the quench model, since the onset of quench differs betweeen the two conductor types.

A lumped-element model of the magnet was created in MATLAB, following the method of Bhattarai *et al* [29]. As can be seen schematically in figure 9, the magnet geometry was divided into 332 radial segments of approximately 2 mm radial thickness each (about 10 turns), so that sufficiently detailed simulations could be performed while still maintaining computational efficiency. The simulation took in to account the different conductor types. The self- and mutual inductances of each coil segment was calculated in a pre-calculation step. The azimuthal and radial currents were calculated for each coil segment on a time-dependent basis, with the current from the power supply as an input variable.

**Figure 10.**
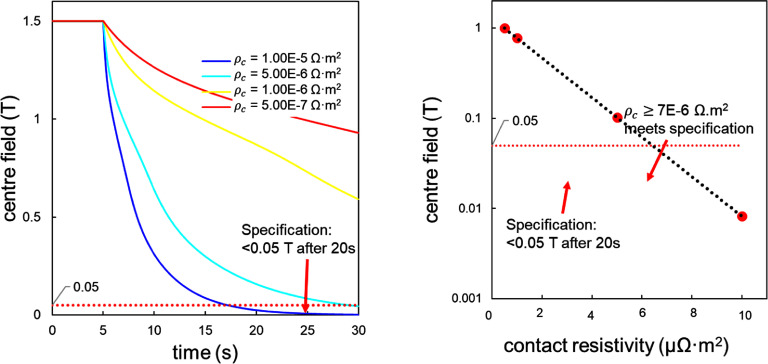
(left) Calculated decline of central magnetic field with time as a function of the contact resistivity during sudden discharge; (right) Calculated central magnetic field after 20 s versus contact resistivity. The assumed resistivity values, represented by red dots, largely overlap with the measured range as shown in figure 12. The black dotted line is a guide to the eye.

To model a sudden discharge, a supply current of 160 A was dropped suddenly to 0 A, and the associated currents and voltages of each coil segment were predicted. These simulations were repeated for different values of contact resistivity, ranging from 0.5 *µ*Ω m^2^ to 10 *µ*Ω m^2^. Results are shown in figure 10, where a sudden discharge was triggered at 5 s. Across the different contact resistivity values studied, central field decline was not perfectly exponential due to heating effects driving some coil segments over the critical current for a short period of time. However, the centre field strength after a sudden discharge was shown to be linearly correlated to the contact resistance. We evaluated the remaining field at 20 s rather than 30 s to ensure compliance with our target <50 mT after 30 s. The results of these simulations are shown in figure 10. We chose a target contact resistivity of 7 *µ*Ω m^2^ to be sure of reaching our goal of <50 mT after 30 s. This was used later during magnet manufacturing to set the filling ratio of conductive particles.

## 8. Epoxy formulation design

From the no-insulation coil model, it was determined the required minimum contact resistivity was 7 *µ*Ω m^2^. From previous work [12], it had been discovered that the epoxy formulation filled with copper particles for electrical conductivity and diamonds exhibited a substantial change in electrical resistivity with temperature. To allow simpler simulation and more predictable coil performance during sudden discharge, we searched for a material with lower temperature sensitivity to electrical resistivity than copper. Ultimately it was decided to replace the copper particles with titanium coated diamonds of the same dimensions as the bare diamonds. Titanium coated diamonds are routinely used in the abrasives industry and readily available. In principle, contact resistivity could therefore be tuned by adjusting the ratio of coated to uncoated diamonds in the epoxy system.

The final coil layup is included below (table 3) for reference. It is important to note the contact resistivity of the coils is necessarily dependent on the choice of the co-wind material and thickness of the epoxy layers between turns. Similarly, for an MRI magnet, the coils must reach their target dimensions and turn count for the magnet to achieve the design uniformity. Selection, validation and consistency of the each of the coil layup components during each coil wind was therefore critical to achieve design specifications, in particular turn-to-turn spacing and contact resistivity.

**Table 3.** Radial thickness of successive components in a turn.

Diamond filled epoxy	12.5 *μ*m
REBCO	95 *μ*m
Diamond filled epoxy	12.5 *μ*m
Ti tape	80 *μ*m

**Figure 11.**
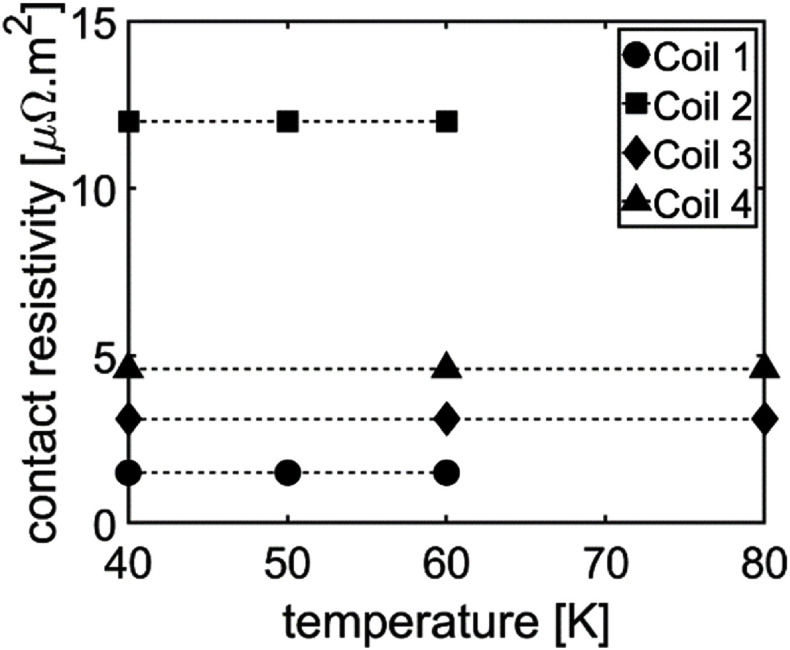
Contact resistivity versus temperature for the four test coils.

**Figure 12.**
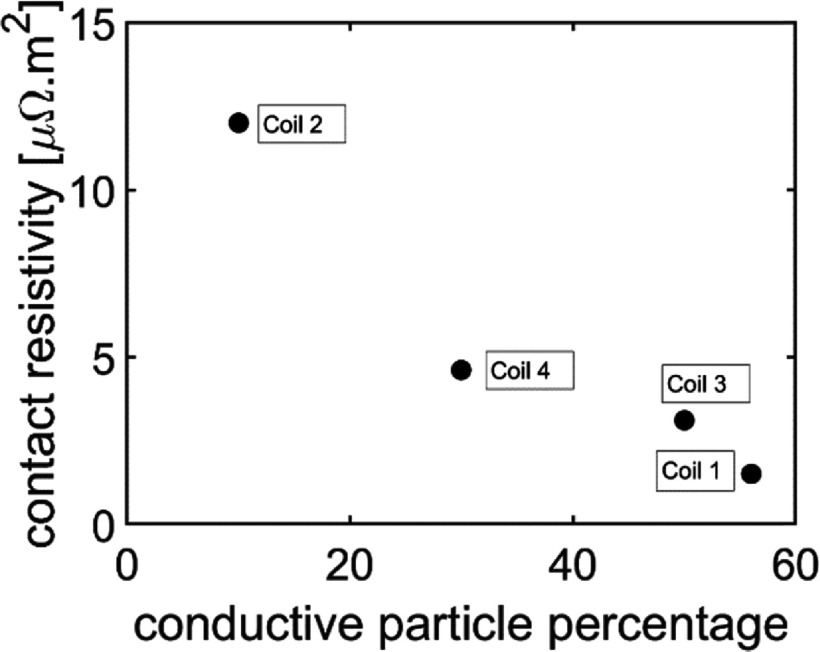
Contact resistivity versus the percentage of conductive particles for the four test coils.

To determine a suitable recipe for the epoxy fillers, we wound a series of four identically sized coils, each with different ratios of coated to uncoated diamonds. The coils were wound according to the layup schedule in table 3, i.e. using 12.5 *µ*m diamonds. Once wound, the coils were conduction cooled and a sudden discharge measurement was performed from 100 A for each coil and the contact resistance calculated. This measurement was repeated for each coil at 40, 50, 60 and 80 K. As can be seen in figure 11, there is minimal temperature-dependence in the contact resistance across the experimental temperature range. The contact resistivity for each of the coils at 60 K is shown in figure 12. Of note, the desired contact resistivity for the magnet (7 *µ*Ω m^2)^) falls inside the range of coated to uncoated diamond ratios tested during this experiment.

We subsequently wound a fifth test coil with the desired ratio of coated to uncoated diamonds and successfully verified the final coil contact resistivity and dimensions.

## 9. Wire quality assurance and conductor placement

**Figure 13.**
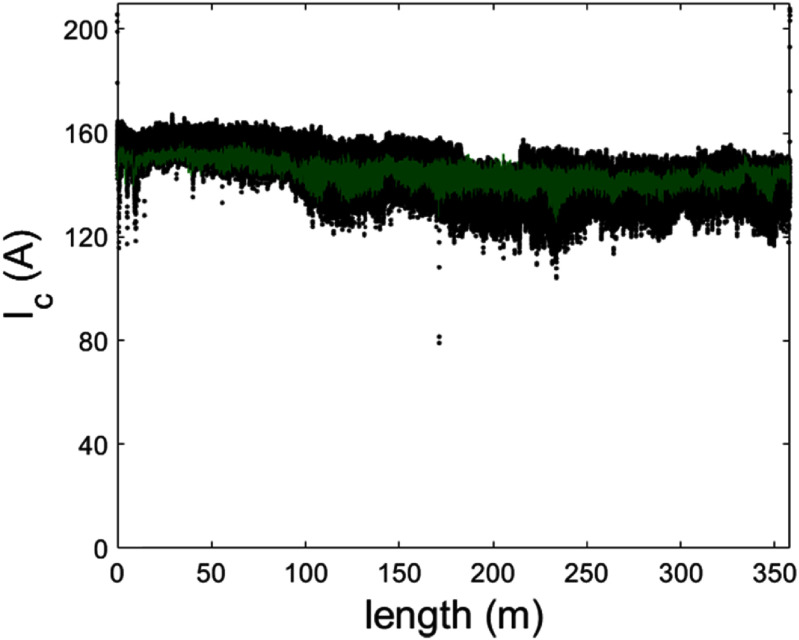
Example of critical current data of a single tape length as provided by the manufacturer (green) and as deduced from DDS magnetization measurements (black).

At the time when the conductor was received, the superconductor vendor characterised their conductor post-manufacture using a 1 m distance resolution. After examination of the as-received conductor, it was found there was a risk defects could be missed using this approach. Therefore, ahead of commencing magnet manufacture, we performed defect detection on the conductor using a dropout detection system (DDS) based around an AC magnetic field measurement system [30]. Since the DDS is uncalibrated, the data was scaled to match the critical current of the supplied scan data from the supplier. In so doing we created an estimate of critical current with high spatial resolution (<2 mm) along the length of the tape. An example showing a length of superconductor with defects overlaying the supplied data is shown in figure 13.

To create defect-free conductor lengths for the magnet, we created a cutting and recombining algorithm. After scaling, the DDS data was ‘cut’ by the algorithm into good and bad sections, also eliminating parts of the conductor with sharp declines in critical current. Each good section represented regions without defects with a minimum length of 50 m. At least 2 m was left between the end of the good section and the start of the bad (i.e. defected) region. We then created a database of good sections noting length, the location specific critical current and conductor type (i.e. GdYBCO or EuBCO).

**Figure 14.**
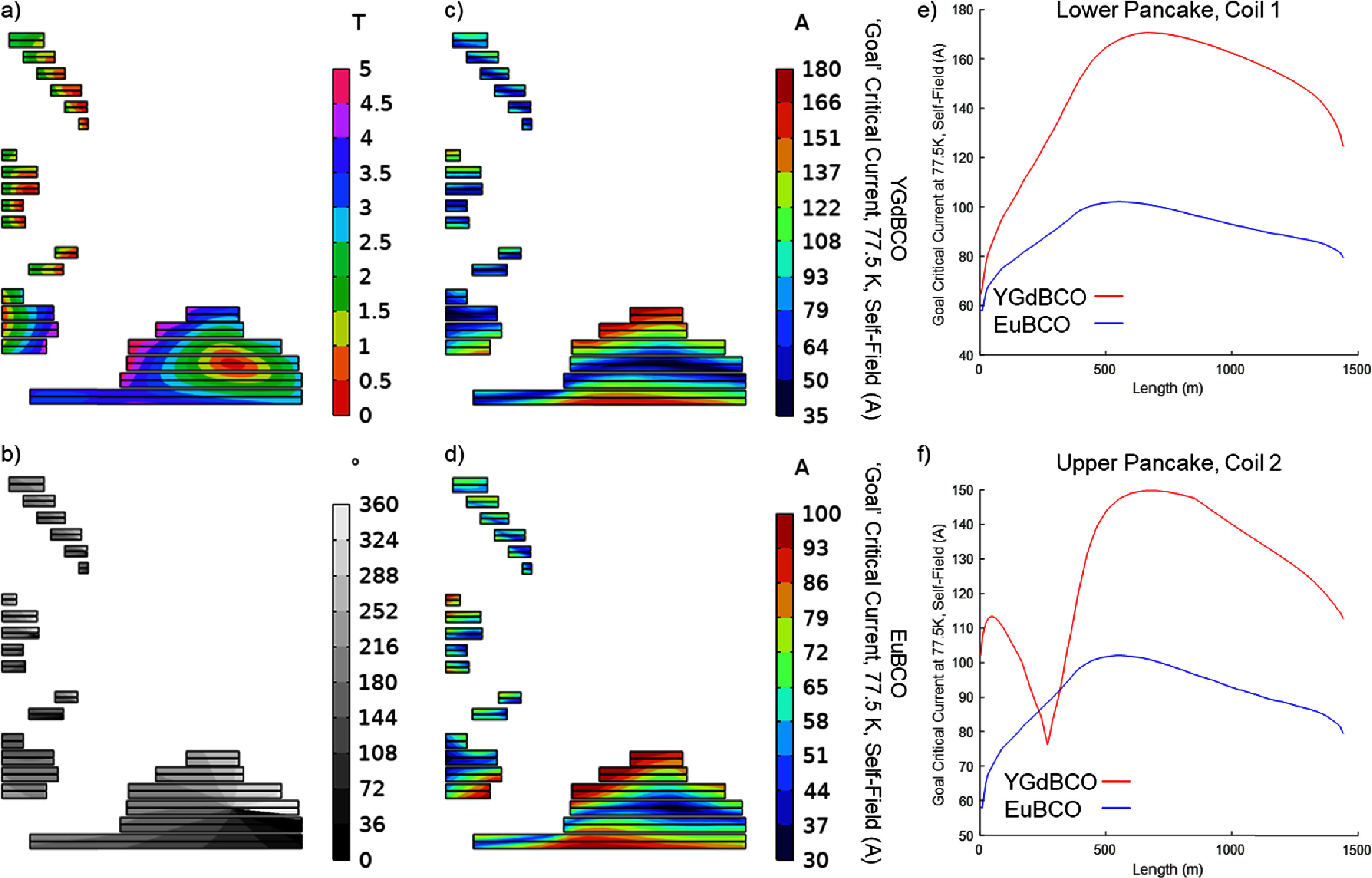
(a) Magnetic field distribution in the windings at 1.5 T centre field; (b) magnetic field angle distribution in the windings at 1.5 T centre field; (c) minimum critical current required at reference conditions if YGdBCO conductor is used; (d) minimum critical current required at reference conditions if EuBCO conductor is used; (e) and (f) minimum critical current required at reference conditions for the conductor in the bottom and top pancake 1 of coil 1 respectively. Magnet geometry plots show the right half of a cross-section and are not to scale. The field angle is defined as 90° when the field is perpendicular to the superconducting face.

Using the previously described measurements for GdYBCO and EuBCO conductors and a simulation of the magnet geometry at the operational temperature and field (figures 4 and 14(a)), it was possible to back-calculate the required critical current of the conductor when measured at 77.5 K self-field to give an in-field critical current of at least 214 A at 30 K everywhere in the magnet (figures 14(c) and (d))—at least 25% above the operating current. This ‘goal’ critical current derived from the simulation could be compared to measurements of the actual conductor to inform placement during winding.

The sorting algorithm would begin by randomly selecting a conductor length and comparing the critical current measurements to the ‘goal’ critical current. If the measurements were below the goal at any location, that conductor would be rejected, and another conductor length would be randomly selected. This continued until all the conductor was placed in a location where it had a high enough critical current to survive in-field operation. The random selection process could be repeated hundreds or thousands of times to optimise for secondary objectives such as minimising the number of joins or minimising the total conductor length.

Figure 14 right top and right bottom show the goal critical current for the two largest coils in the magnet. Due to the variable field and field angle across this coil, the conductor performance requirement changes substantially over the length. The above sorting algorithm would optimise the use of available conductor by choosing tape at the beginning of the winding process with lower quality, reserving high quality tape for the middle of the coil winding, and then selecting medium quality tape to finish the coil. Examples of this are shown later.

**Figure 15.**
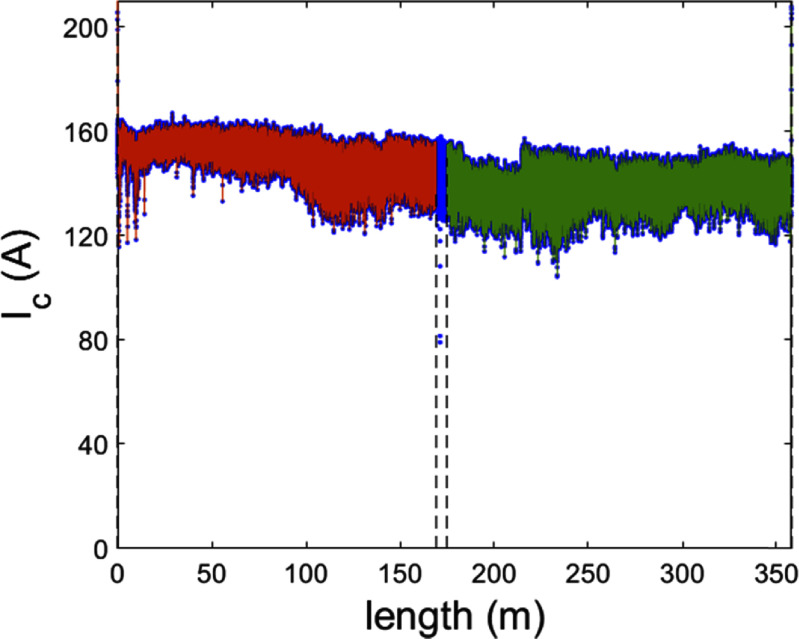
Same DDS data as figure 13 with dashed vertical lines demarcating a section to be cut out and indicating the resulting two tape lengths (red and green).

**Figure 16.**
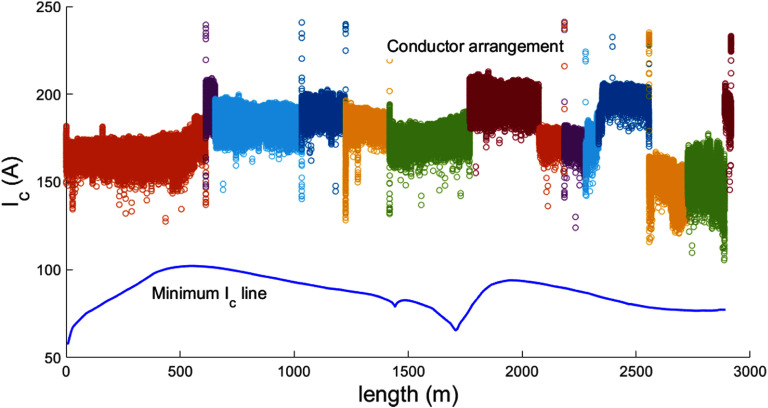
Minimum critical current at the reference conditions of 77 K, self-field required along the length of the conductor for coil 1 (blue line) and the critical current at reference conditions of the selected 14 tape lengths as determined via the DDS system (coloured circles). The solder connections where separate tape lengths are joined cause spurious readings.

An example showing a particular conductor length cut into bad (blue) and good (other colours) is shown in figure 15. Similarly, an example showing a coil assembled from a collection of good sections of conductor is shown in figure 16.

The process of physically collecting the different good sections of conductor required great care in measuring lengths, cutting out defects and then reassembling the conductor in the correct order. This process named respool was time consuming but guaranteed adequate quality superconductor. Connections between the individual tape lengths are made using a bridge-style soldered lap joint with 100 mm overlap on each joint. The assembled single piece of tape was again measured on the DDS to ensure all defects had been removed and the tapes connected in the correct order.

## 10. Coil winding results and size determination for production coils

Following DDS measurement of the assembled pancake conductors, double pancake coils were wound using the pre-prepared conductor. The coils were wound in reverse size order, i.e. small to large. Whilst the electromagnetic design of the magnet called for a particular coil size and turn number, variations in conductor thickness, particularly over several hundred turns meant coils could experience reasonable variation in size or turn count compared to the required amount. To accommodate this, we made small adjustments to coil turn count or coil size for successive coils to keep the magnet’s overall uniformity as designed.

Ahead of winding, the epoxy resin formulation was prepared according to the specification. The mixed epoxy was placed into a pressure pot and applied to the superconductor via a section of narrow flexible tubing. The rate of epoxy flow was therefore controlled by the pressure of the pot and could be varied according to winding speed. Upon completion of winding, the coils were left overnight at room temperature for the epoxy resin to gel and then post-cured at 50 °C for 9 h. After completion of the epoxy curing cycle, the coils were mould released and prepared for testing.

The coils were tested individually and one-at-a-time in a cryogen-free test facility. The facility was sized to accommodate the largest of the coils but was equally capable of testing the smallest coils. The coil testing facility was cooled using a single-stage Cryomech AL 230 cryocooler to a uniform temperature in the range of 39–57 K. Coils were tested to 100 A to verify the absence of any gross coil failure during manufacture and a sudden discharge measurement was performed to allow calculation of the contact resistivity of the coils. A summary of the initial batch of production coils is shown in table 4.

**Table 4.** Actual parameters for wet-wind epoxy-coils.

Coil	Coil ID (mm)	Coil OD (mm)	# turns (−)	Contact resistivity (Ω m^2^)	Inductance (H)	Test temperature (K)
4	400.00	451.44	−240	1.30 × 10^−05^	5.26 × 10^−02^	42
5	544.75	719.00	832	9.62 × 10^−05^	7.73 × 10^−01^	49
6	400.00	464.51	−300	1.91 × 10^−05^	7.97 × 10^−02^	50
8	400.00	458.92	−280	1.88 × 10^−05^	7.03 × 10^−02^	47
9	611.20	671.65	286	5.50 × 10^−05^	1.22 × 10^−01^	50
10	400.00	425.00	−113	7.20 × 10^−06^	1.28 × 10^−02^	46
11	431.60	469.65	189	2.23 × 10^−05^	3.73 × 10^−02^	50
12	460.62	486.50	126	9.48 × 10^−06^	1.93 × 10^−02^	50
13	400.00	426.13	125	6.75 × 10^−06^	1.60 × 10^−02^	41
14	400.00	425.07	112	1.18 × 10^−05^	1.29 × 10^−02^	50
15	400.00	440.00	198	1.67 × 10^−05^	3.79 × 10^−02^	50
16	400.00	439.25	191	2.33 × 10^−05^	3.46 × 10^−02^	50
17	400.00	416.81	80	4.64 × 10^−06^	6.91 × 10^−03^	41
18	328.00	338.55	52	2.52 × 10^−06^	2.40 × 10^−03^	39
19	312.00	336.00	118	6.57 × 10^−06^	1.07 × 10^−02^	50
20	296.00	329.68	165	9.50 × 10^−06^	1.82 × 10^−02^	57
21	280.00	312.30	158	4.95 × 10^−06^	1.61 × 10^−02^	55
22	264.00	299.00	171	6.19 × 10^−06^	1.74 × 10^−02^	55
23	248.00	287.34	191	8.34 × 10^−06^	1.93 × 10^−02^	46

As can be seen in table 1, each of the coils in pack 1, namely coils 1, 2, 3, 5, 7 and 9, require at least 1 km of conductor. Given the large volumes of conductor involved and the large jump in conductor volumes between other coils and the coils from pack 1, we decided to wind the coil pack approximately in size order, testing after each wind to ensure no systematic problems emerged.

**Figure 17.**
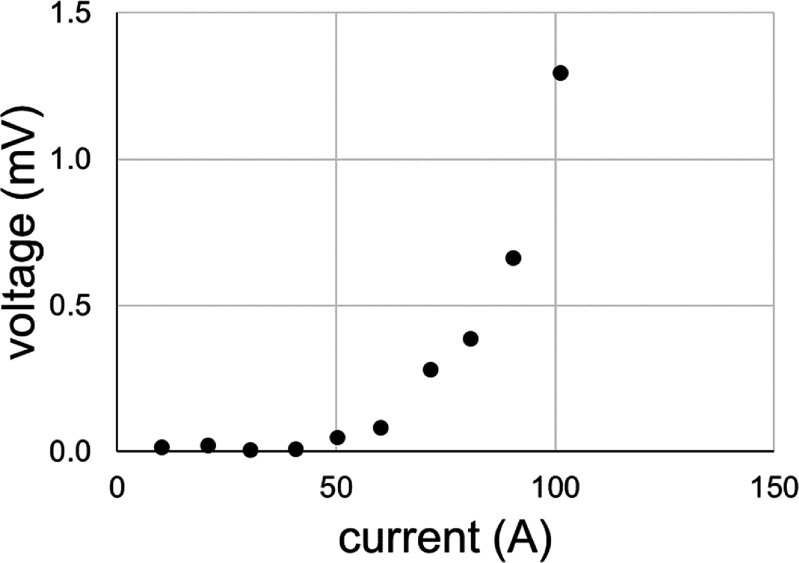
Measured stand-alone voltage–current characteristics of coil 7.

Coils 9 and 5 from this pack were wound without issue as indicated above. Coil 7 was then wound. Following testing of coil 7, we found the coil had undergone significant damage, as can be seen in figure 17. Coil 7 was wound from known defect free conductor and no notable incidents occurred during winding. It was therefore presumed the damage had resulted during cooldown of the coil in the test facility. Since coil 5 had tested satisfactorily, yet the smaller coil 7 had not tested satisfactorily, we were concerned we may be operating near the edge of conductor transverse tensile strength. Compared with smaller coils, the combination of the large radial size of the coils in coil pack 1 and the use of a high-modulus encapsulant may predispose the coils towards failure, especially in combination with the large thermal gradients experienced across the coils during cooldown in the conduction cooled test facility.

Due to the time constraints of the magnet construction, we decided to change to using paraffin wax as an encapsulant. Paraffin wax was chosen as it has been used successfully in a range of both HTS [31] and low temperature superconductor [32] magnets. Whilst we have not specifically analysed the transverse tensile stress of our conductor, nor modelled the stress buildup during coil cooldown, as a low modulus encapsulant it is expected to prevent stress transfer into, and hence delamination of, the ReBCO layer of the conductor during cooldown or warmup. Finally, unlike epoxy encapsulation, it affords the possibility of unwinding the conductor if a coil fails or is found to require repairs following encapsulation.

## 11. Paraffin coils

The decision to infuse the coils with wax had two important implications for the magnet design. Firstly, it was not possible to mix the same filler materials into the wax as had been used for the epoxy. Because wax is much lower viscosity than epoxy when molten, the fillers tended to drop out of suspension with the wax during preparation or were filtered out from suspension during the infusion process. As such, the resulting conductivity of the wax encapsulant could not be assured inside the available time frame. Instead, we decided to provide a 50 *µ*m paper insulation layer between turns. The resulting coil layup is shown in table 5 along with the corresponding epoxy coil layup reproduced from table 3. The mismatch in nominal winding thickness of the two winding techniques was manged by adjusting coil turn count on a coil-by-coil basis to ensure the magnet uniformity remained.

**Table 5.** Comparison between radial thickness of successive components in a turn of paraffin (dry wound) and epoxy (wet wound) coils.

Paraffin coil layup		Epoxy coil layup	
Paper insulation	50 *μ*m	Diamond filled epoxy	12.5 *μ*m
ReBCO	95 *μ*m	ReBCO	95 *μ*m
Ti tape	70 *μ*m	Diamond filled epoxy	12.5 *μ*m
		Ti tape	80 *μ*m

A set of vacuum-tight moulds were then designed to fit around the existing winding mandrels for the magnet. During the infusion process, the coil mould was placed into an oven heated to approximately 70 °C and the coil mould brought under vacuum. The wax was melted in a heating vessel to 80 °C then introduced into the mould via a heated transfer line. Finally, the coil was cooled down to room temperature and removed from the mould. The coil was then mounted into a cryocooled test chamber and tested for critical current retention at ∼50 K and 100 A. The balance of the coils were then wound using the paraffin wax infusion process. Details of the coils are shown in table 6.

**Table 6.** Paraffin production coil parameters.

Coil	ID (mm)	OD (mm)	# turns (−)
1	431.80	744.20	1495
2	535.34	744.70	967
3	542.91	738.28	948
7	576.20	675.88	475

**Figure 18.**
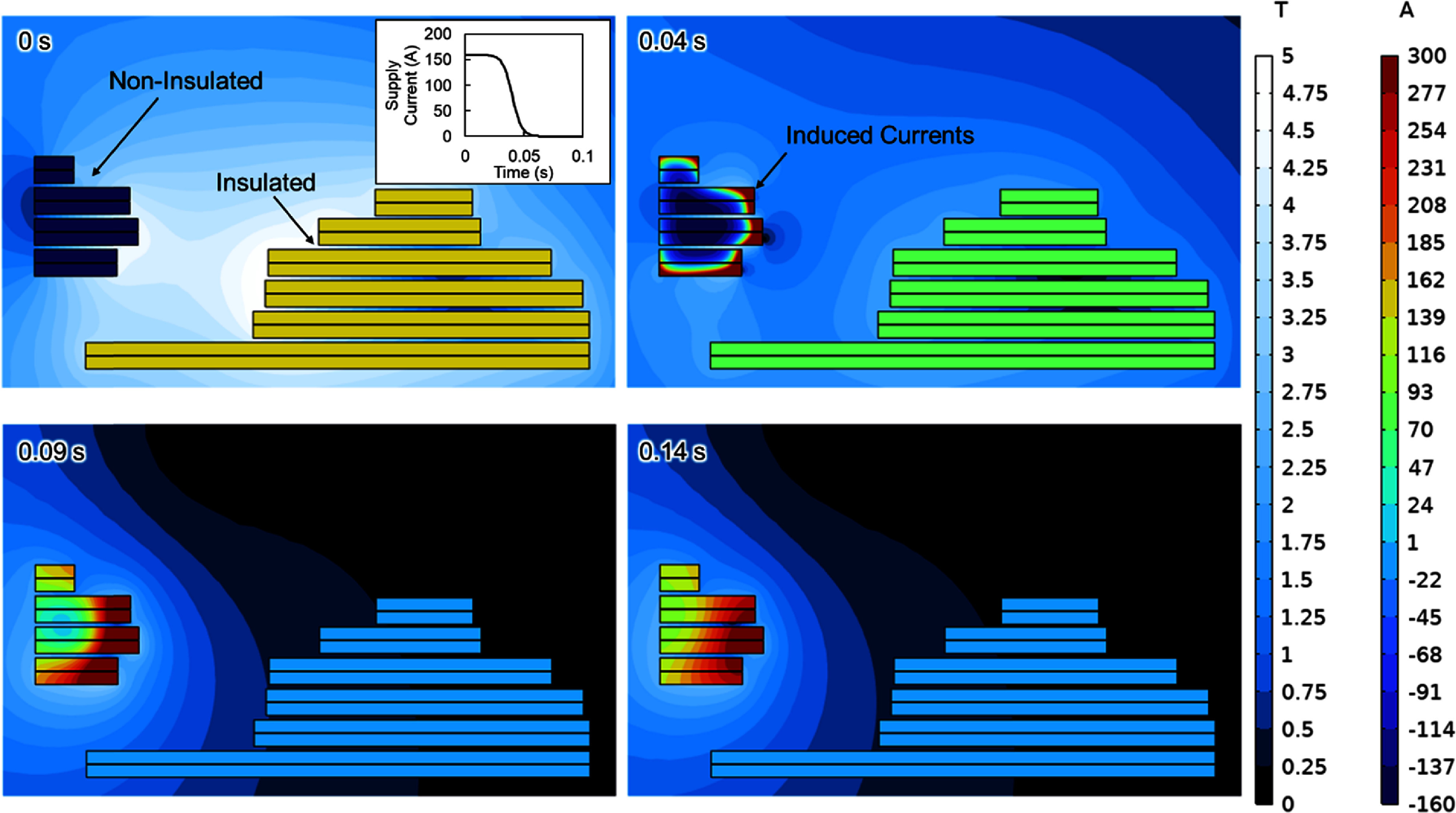
Simulation of head MRI when coil packs 1 are insulated coils and coil pack 2 are NI coils, and magnetic field outside the packs, in a sudden discharge scenario where the supply current falls from 160 A to 0 A, beginning at *t* = 0 s. The ramp down of the current source is shown in the inset figure; *t* = 0 is common between the inset figure and the main figure.

Since the magnet now includes both insulated coils and no-insulation coils, the lumped-element model was re-run to assess how the magnet would perform if the magnet stored energy were allowed to dissipate in the remaining no-insulation coils alone. The contact resistance of the insulated coils was changed to a large value (1 Ωm^2^), so the radial current effectively became zero in the model. When run, the model suggested a concerning outcome: the current decay in the large, insulated coil pack was sufficiently rapid it would induce large current spikes in distant non-insulated coils, driving them significantly over critical and dissipating a large amount of energy quickly in a localised region (figure 18).

The large current and high temperature spikes resulting from protecting the magnet using the remaining no-insulation coils alone suggested the magnet would self-destruct. This meant the magnet needed to incorporate an external dump resistor to provide an additional place for energy to be dissipated in the event of an emergency magnet shut down or quench. A 5 Ω dump resistor was therefore chosen for the magnet, placed in parallel to the coils.

## 12. Magnet protection and monitoring

The instrumentation for the magnet is effectively two separate systems with some overlapping components. One system is the patient and magnet protection for rapidly turning off the magnet in case of danger to the patient or the magnet breaching safe operating thresholds. The other system is a magnet control and health monitor, providing the interface to control the magnet along with monitoring and recording key system parameters.

The protection system has two key requirements. Firstly, it needs to ramp down the field within 30 s of the emergency button push event. Secondly, it needs to detect when the magnet crosses safe operating limits (i.e. temperature or voltage) and initiate a rapid shutdown. As there is a danger of serious injury to personnel, a required performance level of *d* is required according to ISO 13849-1:2023 [33]. As such, the system is designed to be fail-safe, have redundant circuits, and test component integrity after power-on and reset events to achieve the required *d* performance level according to EN ISO 13849-1.

**Figure 19.**
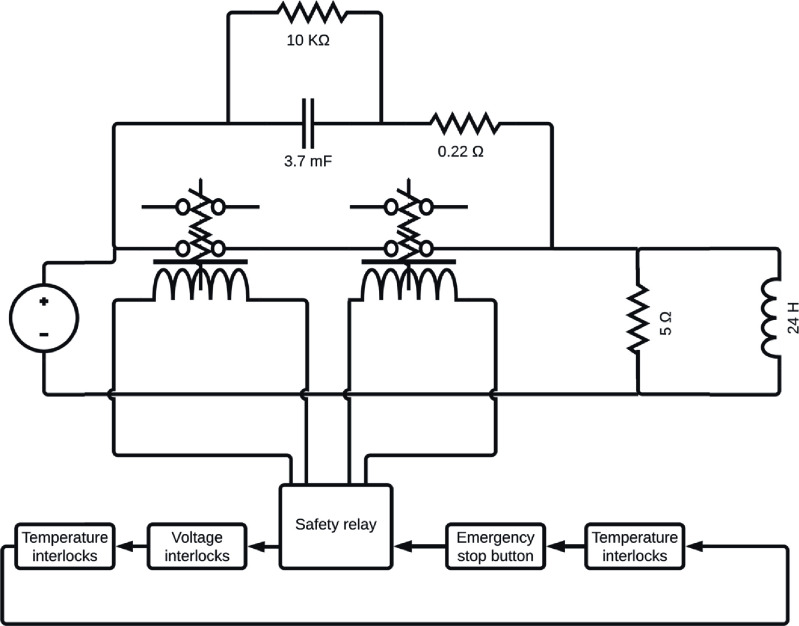
Schematic of the magnet protection system. The magnet is represented by the inductance of 24 H. The 5 ohm resistor provides a dump for the magnet energy when the switches open. The capacitor supresses arcing on the switch contacts by slowing the voltage rise so that the switches can fully open before they experience the full voltage spike from the magnet. The 10 K resistor discharges the capacitor, reducing the stored energy before the switches are allowed to close. The 0.22 Ohm resistor blocks the current spike from the remaining energy in the capacitor when the switches are closed to stop contact welding occurring.

The design used to achieve these requirements is shown in figure 19. There is a pair of redundant normally open contactors connecting the power supply to the magnet. A 5 Ω dump resistor is permanently wired in parallel to the magnet to dissipate magnet stored energy. During normal 160 A operation, 200 mA flows through the resistor. A safety relay drives the contactors with dual interlock circuits running through temperature, voltage, power supply interlock relays and the emergency stop button. If any of the interlocks is open or the emergency stop button is pushed, the safety relay driving the contactors removes power from the contactors, which then open, forcing the full magnet current through the dump resistor, quickly dissipating the energy stored in the magnet. Not shown in figure 19 is the manual reset circuit for the safety relay for checking that both contactors have correctly operated, and no contact welding has occurred.

**Figure 20.**
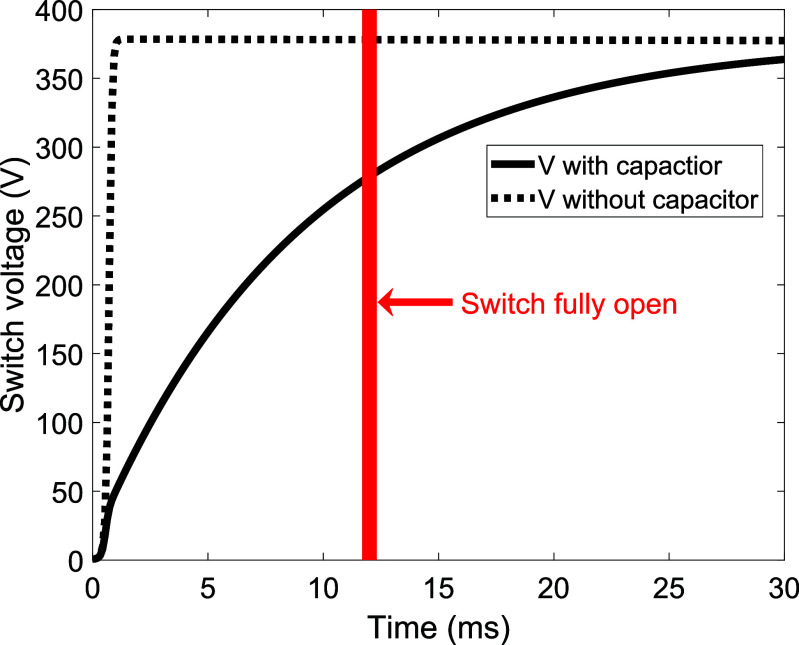
Voltage across the switch that disconnects the power supply from the magnet when the safety system is engaged.

This design makes the contactor for disconnecting the power supply a high-load, critical, active component in the safety system. This contactor needs to handle both the magnet’s 160 A operating current and the high voltage spike generated when switching the magnet’s 24 H inductance (estimated at a peak of 1000 V). To achieve this capability, the GIGAVAC (Carpinteria, USA) HX241 was chosen with a cycle rating of 16 000 under these conditions. However, the contactor is only rated for a load inductance of 27 *µ*H. To provide confidence on the operation of the contactors an arc suppression capacitor was added across the terminals which absorbs the energy during the first 12 ms until the contact has fully opened and can withstand large voltages without arcing. This arc suppression circuit was simulated, showing the voltage at 12 ms after switching is 280 V, comparing favourably to 378 V without the 3.7 mF capacitor (figure 20). The reduction in switching voltage across the contactor improves the expected life of the contactor to the extent contactor failure is unexpected over the lifespan of the magnet.

## 13. Magnet control and monitoring

The magnet control and health monitoring systems are based on a programmable logic controller (PLC) that collects the status and readings from all the monitoring and control devices in the system. These values are logged locally and pushed to a website hosted on the PLC for user monitoring during operation. The temperature monitoring uses 10 Ruthenium Oxide sensors which are read by a pair of Cryogenic Control Systems (Rancho Santa Fe, USA) temperature loggers. Voltages for each coil and each major interconnect in the magnet are monitored with a National Instruments (NI) (Austin, USA) field programmable gate array (FPGA) system with a pair of NI-9209 analogue to digital converters (ADCs). The FPGA is responsible for generation of an interlock signals, when a voltage measured in the magnet exceeds the determined safe threshold for more than 1 s. During magnet ramping, inductive voltages much larger than the safe steady-state voltages are generated across each coil. Therefore, the balance voltage, i.e. the difference between two coil voltages with a correction factor used on the smaller coil’s voltage to correct for any difference in self-inductance, is used to ensure any resistive voltage in a coil is detected. This voltage difference is compared to a pre-set threshold value to determine if the magnet is operating within its safety limits.

Magnet control is achieved via a touch screen interface on the PLC that steps the user through the entire process of taking a warm magnet at atmospheric pressure to a magnetic at full field ready for imaging. At each step the PLC monitors the system to ensure all the requirements have been reached to move to the next step in the process, ensuring the magnet is always operating in safe condition. The PLC controls the turbo pump, cryo-cooler and power supply via RS-232 or MODBUS communication links.

A total of 32 voltage taps and 10 temperature sensors were used on the magnet, all attached with fasteners. The cabling was performed using fine enamel wire routed in PTFE tubing. A temperature sensor was located on each coil pack, opposite from the cryocooler. Similarly, a single voltage tap was run from each pancake, with the voltage difference measured between adjacent coils. When there was a significant space between adjacent coils, for example in the bus between coil packs, an additional voltage tap and temperature sensor was added. During a rapid shutdown event, magnet terminal voltages were expected to reach 400 V. Clamping diodes are used to protect the ADCs during such high-voltage transients. The system has now been thoroughly tested with several complete turn on and turn off cycles without incident. The protection system has been thoroughly testing with in excess of 15 rapid shutdowns including detecting and shutting down magnet in over temperate or voltage events.

## 14. Magnet assembly and first test

**Figure 21.**
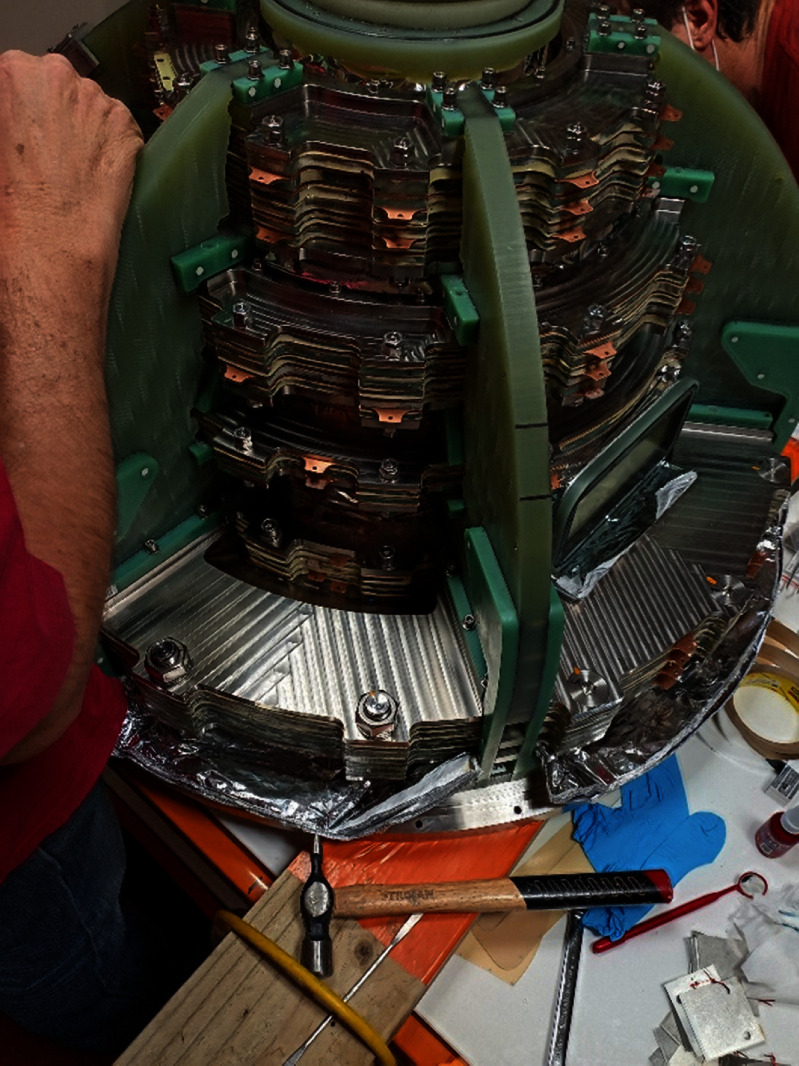
Picture taken during assembly of the cold mass, after mounting the coils in the support structure but before electrical, thermal and instrumentation connections are made. Tabs of the copper cooling plates can be seen extending from the coil packs.

**Figure 22.**
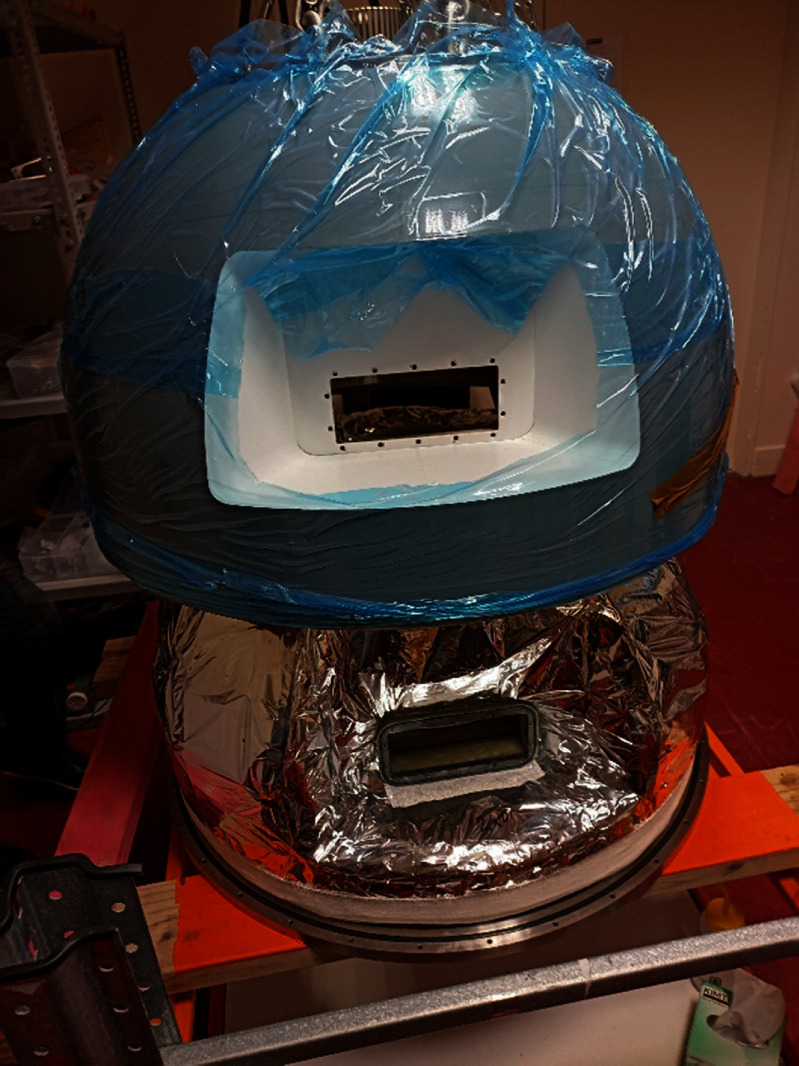
Picture taken during the final phase of magnet assembly showing the cold mass wrapped in MLI and the outer vacuum shell being lowered in place. The rectangular patient view port is prominently in view.

After all the individual coils had been built and tested, magnet assembly started. Each of the five coil packs were assembled in turn and checked for overall thickness. Adjacent double pancake coils were connected in series by ReBCO backed copper strips soldered to the coils with Indium-Bismuth solder. The magnet was then assembled from the five coil packs. The pre-tested voltage tap and temperature sensor wiring assembly was attached to the magnet as discussed in the previous section. The magnet assembly was then wrapped in two blankets of multi-layer insulation. The current bus was then attached to the coils and led back to the cold-head attachment point. Finally, the cryostat lid was placed over the magnet assembly and the connections between the magnet cold bus and the cryocooler completed. Magnet construction can be seen in-progress in figures 21 and 22.

**Figure 23.**
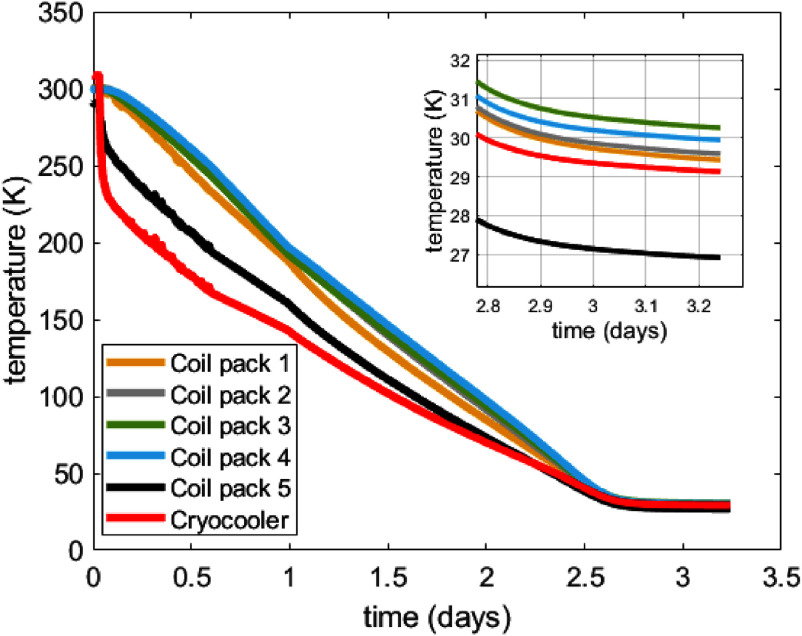
Measured temperature of the cryocooler and coil packs during cool down of the magnet.

The magnet was brought under vacuum and cooled to operating temperature over three days. The cooling curve of the magnet is shown in figure 23. Whilst the cryocooler temperature is ∼1 K lower than the 28 K predicted in section 6, the coil packs match the expected temperatures within ∼1 K suggesting a reasonable match between the thermal design and the as-built magnet.

Once the magnet had reached its operating temperature, 5 A was injected into the magnet using the IECO (Copenhagen, Finland) 250 A power supply, and the correct operation of the magnet safety system verified. The magnet was subsequently ramped to 60 A in a series of 5 A steps. The magnet safety system was successfully tested at 60 A to ensure safe operation.

**Figure 24.**
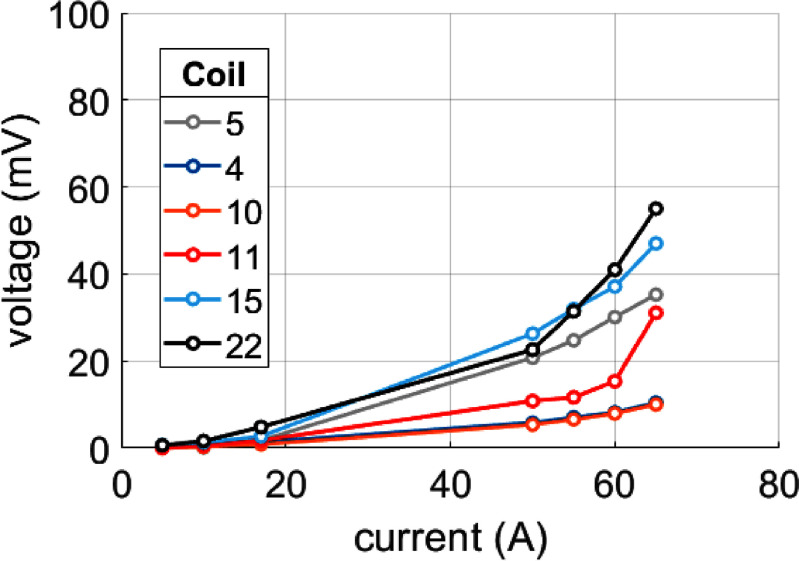
Measured voltages in the six coils that dissipated the most heat during the first test.

During the ramping of the magnet, it became apparent several of the coils had degraded significantly since their stand-alone tests. During the initial ramp to field, the magnet current was held at 50 and 60 A. The voltages across six of the coils indicate significant coil damage. The voltage in each of those coils is shown in figure 24.

At 55 A, the magnet generated 0.487 T, as expected, but did not reach thermal equilibrium and was considered to be in the slow, early stage of thermal runaway. Given that the thermal stability of the magnet was unacceptable at 55 A, relative to a design operating current of 160 A, and significant voltage was observed across some coils as low as 20 A, it was decided to disassemble the magnet and replace as many of the affected coils as possible within the project constraints.

## 15. Magnet assembly and second test

The magnet was disassembled, and new conductor ordered to replace coils 4,5,6,8,10, 15 and 22. Whilst not shown here, coils 6, 8 and 10 were investigated due to coil 4 having failed and showed signs of degradation. Coils 6, 8 and 10 belong to the same coil pack with the negative hoop stress as coil 4. The replacement coils were rewound as paraffin coils and tested as described above. Details of the resulting coils are shown below in table 7.

**Table 7.** Paraffin replacement coil parameters.

Coil	ID (mm)	OD (mm)	# turns (−)
4	401.00	450.57	−234
5	544.75	719.06	746
6	401.00	463.65	−303
8	401.00	675.88	−266
10	401.00	424.40	−110
15	400.00	441.78	189
22	264.00	300.18	163

Following completion of the coil winding and testing process, the magnet was once again reassembled as previously described. The magnet was again cooled to operating temperature over approximately three days.

**Figure 25.**
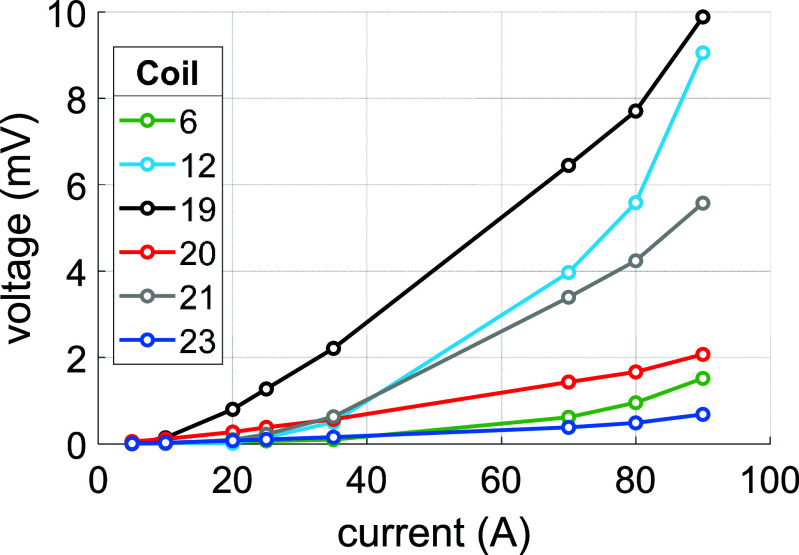
Steady-state voltages of the most-dissipative coils dissipating in the magnet during the second test.

The magnet was ramped to a peak current of 100 A, at which point the magnet protection system initiated safe shut down of the magnet. During the ramp to 100 A, several pauses were made to allow measurement of the coil voltages without any inductive voltage component. The voltage plot for the magnet up to 90 A can be seen in figure 25. As can be seen, magnet performance has been improved since the peak voltage has dropped five-fold at higher current. However, the magnet demonstrates significant voltage drop across several of the remaining epoxy encapsulated coils well below the target operating current of 160 A. After extended testing, it was found the magnet was thermally stable at 81.5 A, corresponding to 0.71 T. The final temperatures and coil voltages are given in tables 8 and 9 respectively. The magnet protection system was found to have operated correctly and safely up to 100 A.

**Table 8.** Steady-state coil pack temperatures at 81.5 A/0.71 T.

Coil pack	Final temperature (K)
1	32.9
2	32.6
3	33.2
4	33.4
5	33.0

**Table 9.** Steady-state coil voltages at 81.5 A/0.71 T.

Coil	Final voltage (mV)
1	0.032
2	0.029
3	0.059
4	0.025
5	0.362
6	1.470
7	0.206
8	0.006
9	−0.021
10	−0.017
11	0.008
12	7.020
13	0.008
14	0.033
15	0.002
16	−0.017
17	0.057
18	0.071
19	9.060
20	1.850
21	4.770
22	0.023
23	0.584

As described in section 2, the magnet is a critical component of a larger grant to manufacture an MRI system. Due to the time constraints of the grant, the decision was made to use the magnet at 0.71 T, analyse the field stability and perform passive shimming.

## 16. Field uniformity and shimming and stability

**Figure 26.**
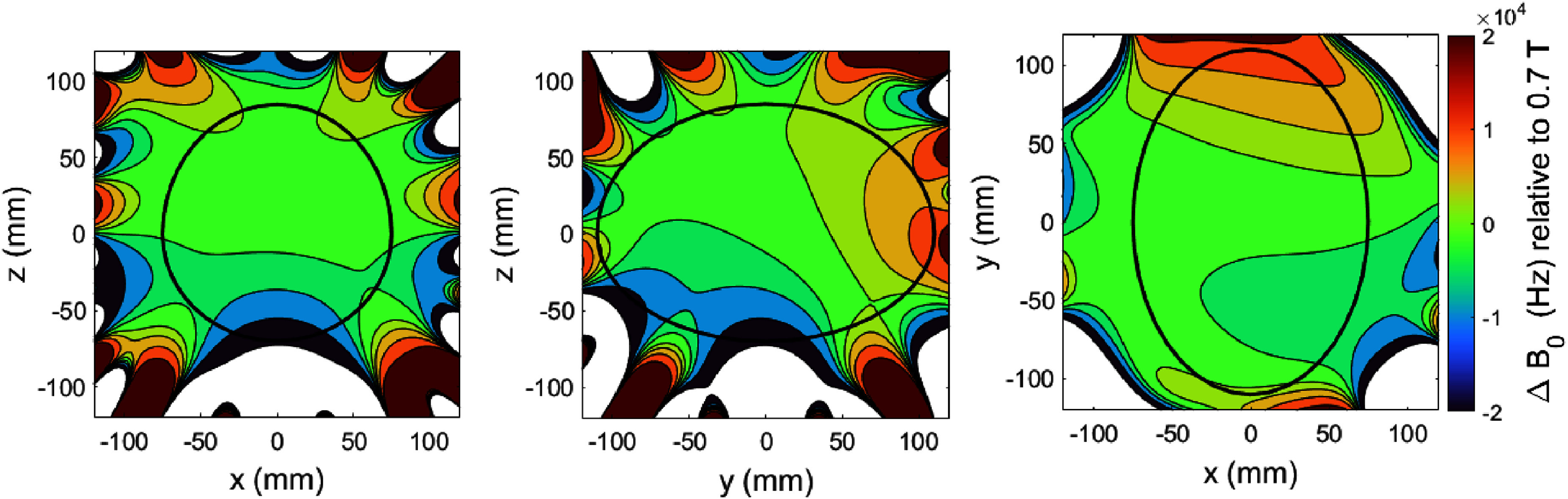
Measured uniformity before passive shimming in Hz relative to the field at magnet isocentre. The black line corresponds to the brain shaped imaging volume used for shimming purposes.

The magnetic field of the magnet was plotted using a high precision Hall probe (Group 3 Technologies, NZ) mounted on a three-axis movement stage (Newark Inc, USA). The field was measured on 20 planes each plane with 24 measurement points located on the surface of an ellipse 200 mm (*y*) × 150 mm (*x*) × 150 mm (*z*) centred about the isocentre of the magnet. The result of the magnetic field plotting is shown in figure 26.

**Figure 27.**
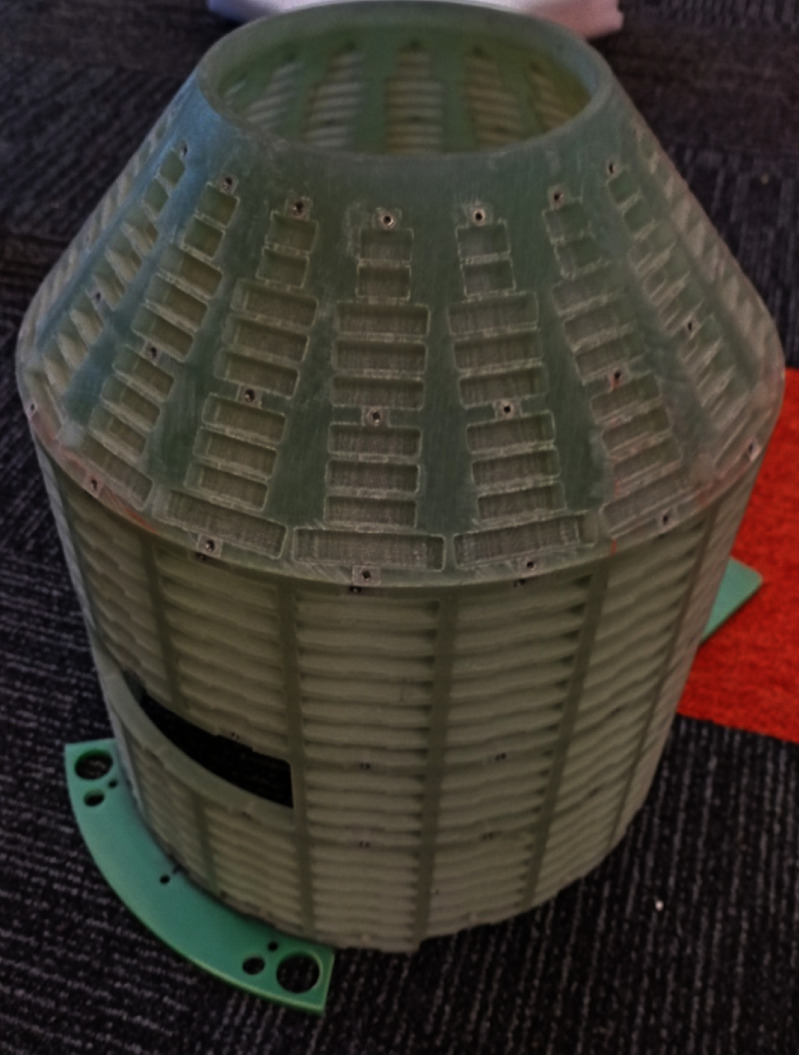
Picture of the passive shim cassette with the view port visible on the lower left.

Unlike most MRI magnets, this magnet has a tapered warm bore. Moreover, to allow for the window through the side of the magnet, there must be a corresponding window through the passive shim cassette. We developed a passive shim cassette for this magnet as can be seen in figure 27 and adapted existing passive shim software to allow for the change in geometry.

**Figure 28.**
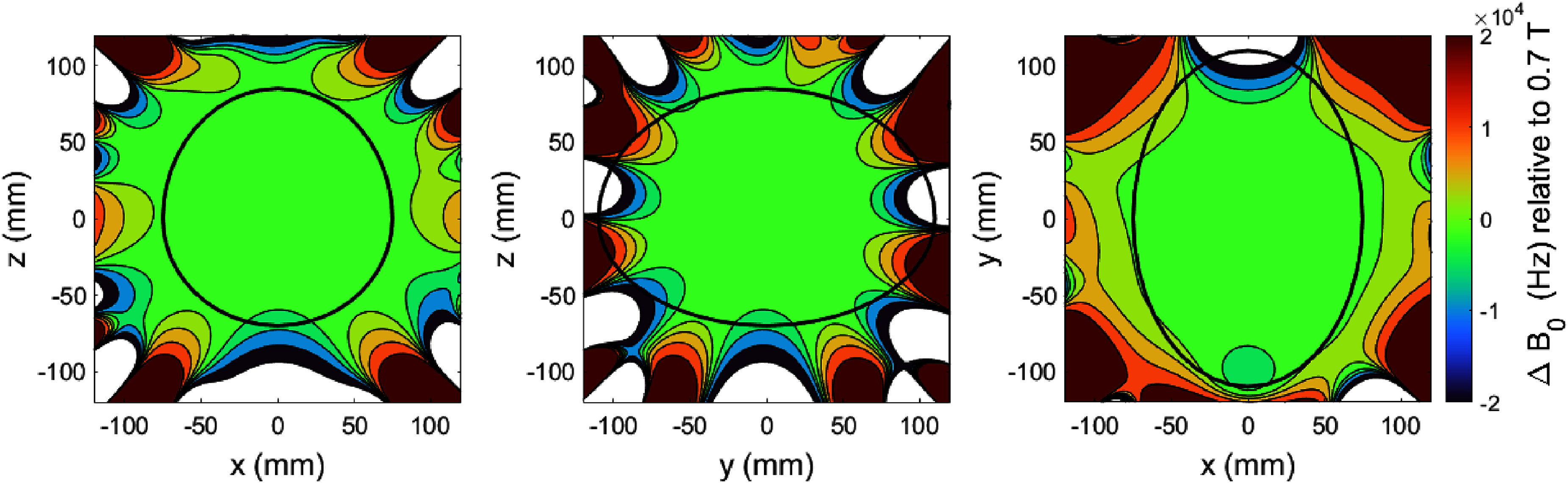
Measured uniformity after passive shimming in Hz relative to the field at magnet isocentre. The black line corresponds to the brain shaped imaging volume used for shimming purposes.

Following measurement of the field over the ellipsoidal imaging volume, we deconvolved the resulting field measurements into a series of orthonormal ellipsoidal functions (analogous to spherical harmonics) and performed passive shimming attempting to minimise each of the ellipsoidal harmonics up to 5th order. The final uniformity can is shown in figure 28, meeting the target field of ±20 kHz peak-to-peak field variation over most of the target volume.

**Figure 29.**
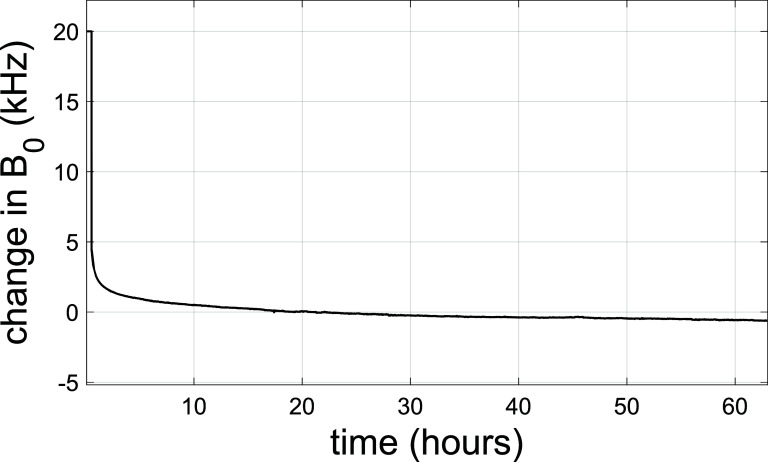
Decay of the central magnetic field *B*_0_ expressed in kHz over 2.5 d after reaching constant current in the magnet.

**Figure 30.**
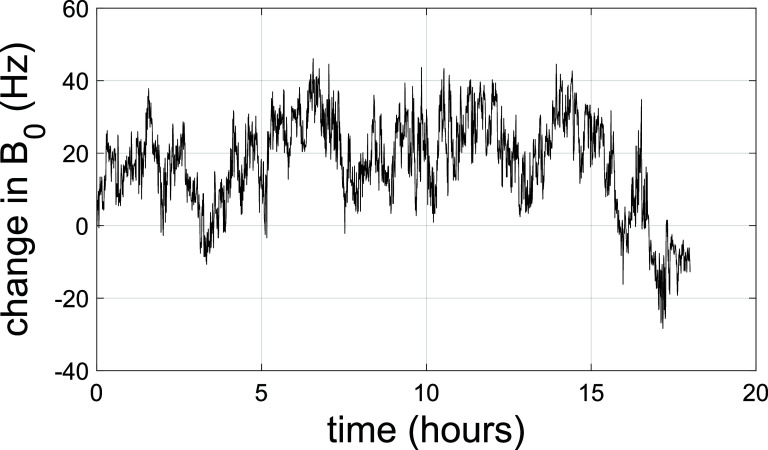
Fluctuation of the central magnetic field *B*_0_ expressed in Hz over 18 h, several weeks after reaching constant current in the magnet. A typical MRI exam is 20 min.

A small proton NMR probe and transceiver circuit was manufactured and mounted in the 3-axis plotting stage. Following ramp to field, the temporal stability of the magnet was measured. The result is shown in figure 29. As a ReBCO magnet, the magnet will experience screening current effects after ramp to field. As the current in most of the coils is well below their critical current, the magnet is predisposed towards a significant and long-lived screening current effects. The continued exponential decay of screening currents is still visible in figure 30 indicating the magnet has still not reached steady state despite the >2.5 d wait period. However, subsequent practical experience with the magnet has not shown this to be a source of concern during imaging and screening current mitigation strategies are not necessary. We have found the magnet stability to be usable for MRI after approximately 8 h.

## 17. Epoxy impregnated coil proposed failure mode

As can be seen in figure 23, during magnet cooldown there are significant thermal gradients between the cryocooler and the coil packs. Our presumption is these thermal gradients are the cause of coil degradation. To first order, we can assume the temperature difference across a coil during cooldown is the same as the temperature difference between the cryocooler and the respective coil pack temperature sensors. The coil pack temperature sensors are located on the opposite side of the magnet to where the cryocooler is attached so this temperature differential can be considered a worst-case scenario. After approximately 0.25 d, there is 72 K difference in temperature between the coldhead (205 K) and coil pack 1 (277 K).

**Figure 31.**
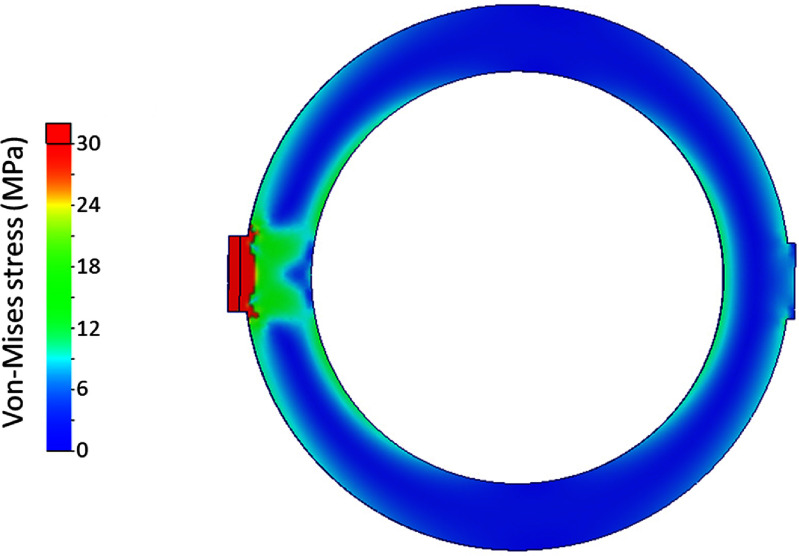
Stress distribution in an isotropic version of coil 5. The right hand side is set to 277 K and the left hand side is set to 205 K. The fixed mechanical boundary position is on the left hand side. There is stress build up evident at the fixed boundary condition which is an artefact of this finite element model; stress plotted in this figure has also been limited to 30 MPa to improve visualisation of the in-coil stress. Out-of-range stress is shown as red.

To produce a lower bound for intra-coil stresses with such a thermal gradient, we performed a multi-physics finite element model using SolidWorks. Here, a simplified version of coil 5 was modelled. The composite structure of the coil detailed in table 3 was simplified to isotropic 316 stainless steel, which has similar thermal contraction properties to the volume weighted thermal contraction rates of the components listed in table 3. The coil model had one side of the coil fixed at 205 K temperature and the other fixed at 277 K. The temperature distribution was then calculated inside the coil. With the cold side of the coil mechanically fixed, the temperature distribution was used to calculate the coil contraction and hence stress. The results are shown in figure 31.

As can be seen in figure 31, stresses of at least 10 MPa are observed far from the fixed boundary condition, in particular near the edges of the coil. Moreover, as detailed in table 3, each coil turn comprises a series of layers namely the ReBCO itself, a titanium co-wind and a filled epoxy encapsulant. When a temperature gradient is imposed across the coil, because each of the layers of the turn are bonded together by the epoxy, the effect of any differential thermal contraction between layers is to create an in-plane shearing force between adjacent layers of the turn. These differential contraction effects would act in addition to the stress calculated in figure 31. Previous studies [34] indicate shear failure in ReBCO conductor at similar (∼11 MPa) values to the calculations presented here. Further study is required to confirm this failure mode, and to set temperature gradient (i.e. shear stress) limits for safe coil operation.

## 18. Summary and discussion

**Figure 32.**
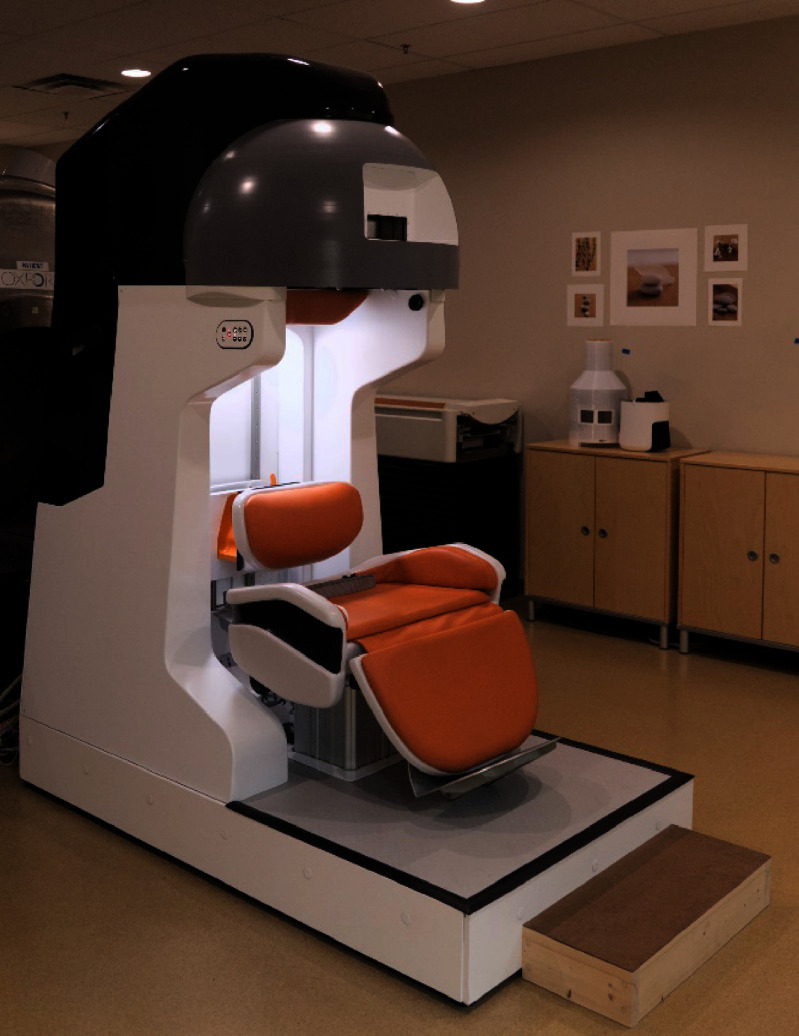
Picture of the magnet and patient interface as installed during system integration.

A 1.5 T ReBCO magnet was designed for brain imaging. The magnet integrated nto the scanner assembly can be seen in figure 32. The magnet was designed with a ±20 kHz variation in magnetic field over an ellipsoidal imaging volume. By allowing the magnetic field to vary more over the imaging volume than regular MRI scanners, it was found to be possible to reduce the length of the magnet to the extent that the patient’s shoulders are outside the magnet.

Within the available space required by the ergonomic constraints of the magnet, the coil configuration was optimized for minimum conductor volume. An initial thermal and mechanical design of the magnet was then performed, leading to a specification for the superconductor, operating temperature and cryocooler performance.

We designed the magnet as a no-insulation style magnet, where the contact resistance between turns was tailored to enable the magnet to be de-energised in less than 30 s in case of emergency. The specific contact resistance of the magnet was created by wet winding using an electrically conductive epoxy. We filled the epoxy with precisely sized diamonds to allow precise control of turn-to-turn spacing in the superconducting coils. By tuning the ratio of titanium coated (i.e. conductive) and uncoated (i.e. non-conductive) diamonds in the epoxy, it was found contact resistance could be controlled to achieve the required magnet de-energisation time.

To commission the coil winding process, a series of small coils were wound and tested. These coils allowed us to determine the ratio of conductive to non-conductive diamonds needed to achieve the target de-energisation time. During the commissioning process, the coils were individually tested in a conduction cooled test facility to verify critical current retention and determine contact resistance. The test coils were thermally cycled during testing.

Following wire quality assurance, we wound and individually tested 19 of the required 23 coils, in approximately ascending size order. During winding of coil pack 1 coils, we experienced low critical current and *n*-value in one of these epoxy encapsulated coils. To manage the risk of subsequent large coil failure, we switched to wax-impregnated and hence fully insulated coils for the remaining coil pack 1 coils. This necessitated the addition of an external dump resistor to safely manage the magnet’s stored energy during emergency shut down.

Following completion of the coil winding, the magnet was assembled during 2022 and tested. During ramps up to 87 A, it was found several of the smaller epoxy-encapsulated coils had become damaged between their stand-alone test and operation of the assembled magnet. This damage caused both noticeable voltage in these coils and temperature rise in the magnet. The magnet was thermally stable at approximately 50 A, rather than the target 160 A.

The magnet was then stripped down and the damaged epoxy coils replaced with paraffin encapsulated coils. The magnet was then rebuilt and operated. Again, coil damage became apparent in two small epoxy coils, this time limiting performance to 90 A/0.8 T. For programmatic reasons, it was decided to stop working on the magnet and ship it to our collaborators as a 0.71 T magnet. Subsequent analysis leads us to believe the cause of the damage in the epoxy coils is because of the large thermal gradients experienced in the magnet during cooldown. Further publications will explore this failure mode in more detail and continue to develop this coil impregnation method.

The uniformity and stability of the resulting magnet were assessed post-construction. The field uniformity met the required ±20 kHz peak-to-peak metric following passive shimming. The magnet stabilises sufficiently within 8 h for MRI, and stable to within the power supply specifications within 24 h. The magnet has now been successfully deployed at our collaborators and integrated into an MRI scanner. Imaging performance will be detailed in a subsequent publication.

## Data Availability

The data cannot be made publicly available upon publication because they are not available in a format that is sufficiently accessible or reusable by other researchers. The data that support the findings of this study are available upon reasonable request from the authors.
